# Adaptation Strategies to Improve the Resistance of Oilseed Crops to Heat Stress Under a Changing Climate: An Overview

**DOI:** 10.3389/fpls.2021.767150

**Published:** 2021-12-15

**Authors:** Muhammad Ahmad, Ejaz Ahmad Waraich, Milan Skalicky, Saddam Hussain, Usman Zulfiqar, Muhammad Zohaib Anjum, Muhammad Habib ur Rahman, Marian Brestic, Disna Ratnasekera, Laura Lamilla-Tamayo, Ibrahim Al-Ashkar, Ayman EL Sabagh

**Affiliations:** ^1^Department of Agronomy, University of Agriculture, Faisalabad, Pakistan; ^2^Horticultural Sciences Department, Tropical Research and Education Center, Institute of Food and Agricultural Sciences, University of Florida, Homestead, FL, United States; ^3^Department of Botany and Plant Physiology, Faculty of Agrobiology, Food and Natural Resources, Czech University of Life Sciences Prague, Prague, Czechia; ^4^Department of Forestry and Range Management, University of Agriculture, Faisalabad, Pakistan; ^5^Department of Agronomy, Muhammad Nawaz Shareef University of Agriculture, Multan, Pakistan; ^6^Crop Science Group, Institute of Crop Science and Resource Conservation (INRES), University Bonn, Bonn, Germany; ^7^Department of Plant Physiology, Slovak University of Agriculture, Nitra, Slovakia; ^8^Department of Agricultural Biology, Faculty of Agriculture, University of Ruhuna, Kamburupitiya, Sri Lanka; ^9^Department of Plant Production, College of Food and Agriculture, King Saud University, Riyadh, Saudi Arabia; ^10^Agronomy Department, Faculty of Agriculture, Al-Azhar University, Cairo, Egypt; ^11^Department of Field Crops, Faculty of Agriculture, Siirt University, Siirt, Turkey; ^12^Department of Agronomy, Faculty of Agriculture, Kafrelsheikh University, Kafr El-Shaikh, Egypt

**Keywords:** antioxidants, CRISPR/Cas9 technology, heat stress, oilseeds, omics technology, signaling, smart technologies, tolerance

## Abstract

Temperature is one of the decisive environmental factors that is projected to increase by 1. 5°C over the next two decades due to climate change that may affect various agronomic characteristics, such as biomass production, phenology and physiology, and yield-contributing traits in oilseed crops. Oilseed crops such as soybean, sunflower, canola, peanut, cottonseed, coconut, palm oil, sesame, safflower, olive etc., are widely grown. Specific importance is the vulnerability of oil synthesis in these crops against the rise in climatic temperature, threatening the stability of yield and quality. The natural defense system in these crops cannot withstand the harmful impacts of heat stress, thus causing a considerable loss in seed and oil yield. Therefore, a proper understanding of underlying mechanisms of genotype-environment interactions that could affect oil synthesis pathways is a prime requirement in developing stable cultivars. Heat stress tolerance is a complex quantitative trait controlled by many genes and is challenging to study and characterize. However, heat tolerance studies to date have pointed to several sophisticated mechanisms to deal with the stress of high temperatures, including hormonal signaling pathways for sensing heat stimuli and acquiring tolerance to heat stress, maintaining membrane integrity, production of heat shock proteins (HSPs), removal of reactive oxygen species (ROS), assembly of antioxidants, accumulation of compatible solutes, modified gene expression to enable changes, intelligent agricultural technologies, and several other agronomic techniques for thriving and surviving. Manipulation of multiple genes responsible for thermo-tolerance and exploring their high expressions greatly impacts their potential application using CRISPR/Cas genome editing and OMICS technology. This review highlights the latest outcomes on the response and tolerance to heat stress at the cellular, organelle, and whole plant levels describing numerous approaches applied to enhance thermos-tolerance in oilseed crops. We are attempting to critically analyze the scattered existing approaches to temperature tolerance used in oilseeds as a whole, work toward extending studies into the field, and provide researchers and related parties with useful information to streamline their breeding programs so that they can seek new avenues and develop guidelines that will greatly enhance ongoing efforts to establish heat stress tolerance in oilseeds.

## Introduction

Oilseeds are ranked fourth in important food commodities after cereals, vegetables and melons, and fruits and nuts, and they occupy about 213 Mha of the world's arable land (OECD-FAO, 2020). However, the utilization and demand of oil crops continuously increases due to high population pressure, vagaries in dietary choices, cumulative global affluence, and the need for more renewable bio-products (Villanueva-Mejia and Alvarez, 2017). Vegetable oil is used as a biofuel, so it has a great future as an essential energy source (Lu et al., 2011). Factually, the primary sources of vegetable oils are oilseed crops, including rapeseed, soybean, cotton, peanut, palm oil, and sunflower (Abiodun, 2017), which are used in human diets as salad dressings, oil, margarine, frying oil, and numerous other products. Due to their specific chemical and physical properties, vegetable oil is an important feedstock used to produce multiple industrial materials, including promising applications such as biofuel and constituting an alternative to petroleum derivatives (Lu et al., 2011). Oilseed crops are a significant source of animal (Ponnampalam et al., 2019) and human nutrition (Rahman et al., 2018a) and industrial products (Liu et al., 2018a), and biodiesel production (Mohammad et al., 2018) has been increasing day by day. The quality and consumption of oilseed crops have been improved through different genetic engineering techniques (Tan et al., 2011).

Numerous environmental stresses affecting plant growth and development have induced grave anxiety in the context of potential climate change. Across the globe, contemporary agriculture is facing unprecedented environmental pressure and stress due to climatic variability (Argosubekti, 2020). Plants' growth in open environments faces several challenges, including heat, drought, cold, waterlogging, and salinity (Ashraf et al., 2018). Elevated temperature is one of the major concerns for the world as different models have predicted the rise of carbon dioxide (CO_2_), causing an increase in the ambient temperature leading to global warming (NOAA, 2017), which would have severe consequences on agriculture production systems across the globe. The Intergovernmental Panel on Climate Change (IPCC) estimates that the global ambient temperature will increase by 1.5°C from 2030 to 2052 (IPCC, 2018). Temperature-induced heat stress is articulated as the shift in air temperature exceeding the threshold level for an extended period that could cause injuries or irreversible damage to crop plants in general (Teixeira et al., 2013). Therefore, heat stress has proven to be a great menace and ever-looming threat to fruitful crop production around the globe (Hatfield and Dold, 2018; Tariq et al., 2018). The consequences of global climate change and spatial, temporal, and regional patterns are of considerable concern in agriculture production (Porter and Moot, 1998). Heat stress speeding up crop growth and not allowing the proper completion of crop growth stages results in immature development (Rahman et al., 2018a), perturbing carbon assimilation. This is an urgent matter, given that the geographical distribution of plant species depends to a large extent on their adaptation to different temperature zones (Keller and Seehausen, 2012).

Additionally, the world population is expected to reach 9 billion by 2050. Agriculture production needs to be enhanced up to 70% regardless of climate change and its impacts on agriculture (Rahman et al., 2018b). However, all the growth stages in plants are affected adversely by heat stress right from germination to growth and development, reproductive phase, seed yield (Hasanuzzaman et al., 2013; Ahmad et al., 2016), and seed quality in oilseed crops (Ahmad et al., 2021a). The rise in global temperature will ultimately damage the ecosystem comprehensively (Kanojia and Dijkwel, 2018). Specifically, heat stress is a severe threat to oilseed crops as it impairs the production and quality of the yield; for example, the seed yield decreased up to 39% in camelina and 38% in canola under elevated temperature scenarios (Jumrani and Bhatia, 2018; Ahmad et al., 2021b).

The temperature fluctuations have made it imperative to develop climate-resilient varieties that display better adaptability for growth under varied environmental conditions (Bhat et al., 2021). However, achieving this objective will be complicated by the fact that the performance of oilseeds may be hampered by environmental impacts related to climate change and the associated increase in pests and diseases, which are likely to become more challenging in the near future (Jaradat, 2016; Rahman et al., 2019). Therefore, hypothetically, several options can be used to achieve improvements in seed yield and related traits (either alone or in combination), increase seed oil content, or reduce seed yield losses due to abiotic stresses, including high temperature at the sensitive crop stage (Valantin-Morison and Meynard, 2008). The resilience of oilseed crops under heat stress is led by conventional breeding techniques, including hybridization, artificial selection, and induced mutagenesis; though, these methods are complicated due to the polyploid nature of oil crops and require extensive time and labor investments to accomplish (Yang et al., 2017). In the coming decades, the growing demand for oilseeds can be achieved by using advanced molecular breeding techniques such as complementary breeding tools, which would be very useful to accelerate all crop improvement programs to produce climate-resilient crops. While transgenic approaches have so far been successfully used in oilseeds to improve a wide range of traits (Meesapyodsuk et al., 2018; Na et al., 2018; Shah et al., 2018; Kim et al., 2019; Wang et al., 2019), only a small number of these devices have made it to the market due to poor public perception as well as the disproportionately high cost and length of existing regulatory processes (Mall et al., 2018). Therefore, in this review, we aim to analyze recent results on the response and tolerance to heat stress at the cell, organelle, and whole plant level and describe the numerous approaches used to increase heat tolerance in oilseed crops.

## Heat Stress and Its Threshold in Oilseeds

In general, the threshold level is defined as a point after which some irreversible changes might occur. Therefore, the threshold level of heat stress is the moment after which plants lose their membrane stability. The scorching impact of high-temperature stress can be defined by the duration of exposure, the intensity of focus, and the degree of elevated temperature. Temperature limits of 35°C are considered heat stressors in tropics and subtropics (Bita and Gerats, 2013; Awais et al., 2017a; Ahmad et al., 2021a; Waraich et al., 2021a); however, temperatures above 25°C are thought to be stressors in rabi (winter) crops (Wahid et al., 2007; Abbas et al., 2017). The impact of high-temperature stress and the threshold temperatures of important oilseed crops at different growth stages is presented in Table 1.

**Table 1:** Effect of heat stress in different oilseed crops at different growth stages.

**Oilseed**	**Heat stress/duration**	**Impact on plant**	**Growth stage**	**References**
Soybean (*Glycine max* L.)	42/34°C	Length between nodes and internodes decreased.	Seed filling	Allen Jr et al., 2018
	45°C/6 days	Chlorophyll content and yield	Reproductive phase	Khan et al., 2020
	38°C/8 hours	Decreased seed production	The appearance of the first flower	Cohen et al., 2021
	40°C/14 days	Reduced seed production and yield	Seed fill	Djanaguiraman et al., 2011
	42/28°C	Leaf weight, stomatal density, photosynthesis, and chlorophyll fluorescence	Reproductive phase	Jumrani et al., 2017
Sunflower (*Helianthus annuus* L.)	25°C/7 days after first anthesis to physiological maturity	Decreased the size of the embryo	Reproductive stage	Chimenti et al., 2001
	38°C/3 weeks	Increased lipid peroxidation and hydrogen peroxide content	Reproductive stage	Razik et al., 2021
	35°C/7 days	Decreased the seed weight per plant, decreased oil content	Seed fill stage/reproductive stage	Rondanini et al., 2003
	33°C/6 weeks	Decreased the leaf growth in sunflower	Vegetative	De la Haba et al., 2014
Canola (*Brassica napus* L.)	35°C/14 days	Reduction in gas exchange and water relations	Reproductive stage	Ahmad et al., 2021c
	37°C/2 days	Seed photosynthesis machinery, impairment of carbohydrates incorporation	Reproductive stage	Huang et al., 2019a
	35°C/7 days	Abnormal vegetative growth	Reproductive stage	Chen et al., 2021a
	32°C/7 days	Female reproductive organs are more sensitive than male reproductive organs	Reproductive stage	Chen et al., 2021b
	28°C/10 days	Reduced water relation and seed yield	Reproductive stage	Waraich et al., 2021b
Groundnut (*Arachis hypogaea* L.)	34°C/6 days	Reduction in number of pegs and pods	Reproductive	Prasad et al., 2000
	40°C/6 days	90% reduction in pod formation	Micro-sporogenesis	Prasad et al., 2001
	40°C	The photochemical efficiency of PSII decreased	Vegetative	Yang et al., 2013
	41°C/18 days	Fatty acid profile	Flowering	Lwe et al., 2020
Cotton (*Gossypium hirsutum* L.)	40°C/6 h	Reduction of photosynthetic material, total soluble sugars, and proline content	Reproductive stage	Mohamed and Abdel-Hamid, 2013
	45°C	Reduction in photosynthesis and cell membrane stability	Reproductive stage	Saleem et al., 2021
	38 and 45°C/1 week	Increased lipid membrane damage through increased malondialdehyde (MDA)	Reproductive stage	Sarwar et al., 2019
Castor bean (*Ricinus communis* L.)	35°C/13 days	Effect biomass production	Germination stage	Ribeiro et al., 2014
	35°C/7days	Heat shock proteins	Germination stage	Ribeiro et al., 2015
Linseed (*Linum usitatissimum* L.)	>30°C/7 days	Pollen viability	Reproductive stage	Gusta et al., 1997
	42°C/ 1 day	Gene expression	Reproductive stage	Saha et al., 2021
Camelina (*Camelina sativa* Crantz)	25–35°C/3 h in a day	Reduced photosynthetic rate	Reproductive stage	Carmo-Silva and Salvucci, 2012
	35°C/14 days	Reduction in gas exchange and water relations	Reproductive stage	Ahmad et al., 2021a
	35°C/10 days	Oxidative damage	Reproductive stage	Ahmad et al., 2021b
	35°C/14 days	Photosynthetic rate and water status decreased	Reproductive stage	Ahmad et al., 2021c
	32°C/12 days	Reduced growth rate and gas exchange	Reproductive stage	Waraich et al., 2021a

## Heat Stress Sensing and Signaling

A healthy plant needs a compact and robust network of interconnected systems that responds rapidly to stimuli, initiates metabolic responses, and exhibits unique plasticity to adapt to adverse conditions. Heat stress can affect plant functioning in various ways by destabilizing membrane fluidity, multiple proteins, transport systems, enzyme efficiency, RNA stability, and de-polymerization of the cytoskeleton (Hasanuzzaman et al., 2013). The adaptation process to stress is complex and occurs mechanistically through genes, metabolites, and proteins that are collectively involved in many regulatory pathways. The initial step of stress perception involves molecular or structural changes through which a signaling cascade is established, leading to membrane fluidity responses, adaptive changes in proteins, and alteration of DNA and RNA sequences (Lohani et al., 2020). The initial site of stress sensing is mostly the plasma membrane that stimulates the activation of Ca^+2^ channels in the plasma membrane resulting in oscillations of the cytosolic Ca^+2^ level. Ca^+2^ acts as a secondary messenger, and signals rely on Ca^+2^ sensors and others such as calcineurin B-like proteins (*CBLs*), calmodulin (*CaMs*), calmodulin-like proteins (CMLs), calcium-dependent protein kinases (CDPKs/CPKs), G protein-coupled receptors (GPCR), mitogen-activated protein kinase (MAPKs), pyrabactin resistance 1-like (PYR/PYL) protein, matrix metalloproteinases (MMPs), and other enzymes. For the most part, this mechanism of calcium detection has been elucidated in several models and also in oilseed plants.

### Calmodulin and Calmodulin-Like Proteins

CaM and CML-containing helix-loop-helix EF-hand domains are a family of Ca^2+^ sensors in plants and control downstream targets based on Ca^2+^ fluctuations (Lohani et al., 2020). Eighteen CAMTAs have been identified in *B. napus*, the maximum of any plant species reported to date (Rahman et al., 2016). Diversified expression of these BnaCaM/CML genes indicated significant roles in different tissues in response to stress conditions, including heat stress. It was critical in the upregulation of heat stress tolerance (He et al., 2020). These proteins played essential roles in 13 metabolic processes and cellular responses, including protein biosynthesis, carbohydrate metabolism, protein folding, signal transduction, carbon assimilation and assembly, cell cycle, energy pathway, cell defense and rescue, nitrogen metabolism, lipid metabolism, transcription regulation, amino acid metabolism, and secondary metabolite biosynthesis (Wang et al., 2012).

### Calcineurin B-Like Proteins

In contrast to calmodulin, which regulates several proteins, calcineurin B-like proteins are apparently linked to calcineurin B-like protein kinases (CIPK) or SNF1-related protein kinases (SnRK3) (Chen et al., 2012). The structural composition of calcineurin B-like interacting protein kinases contains an N-terminal kinase catalytic domain. This junction domain links it to the highly variable C-terminal regulatory part (Chaves-Sanjuan et al., 2014). The C-terminal regulatory environment consists of the FISL motif with a unique 24 amino acid stretch, essential for the CBLCIPK binding (Albrecht et al., 2001). Yuan et al. (2014) stated the description of CBL and CIPK genes in *B. napus* and revealed the presence of 23 CIPKs and 7 CBLs. Interaction studies of BnCBL1-BnCIPK6 protein were established by bimolecular fluorescence complementation (BiFC) and its regulation under stressed conditions in *B. napus* (Chen et al., 2012).

### Calcium-Dependent Protein Kinase

Calcium-dependent protein kinases act as a third component of the Ca^2+^ sensing apparatus in plants, functioning as a responder to various sensors with the ability to self-modify authorization through the action of various enzymes (Chen et al., 2012), making calcium-dependent protein kinases very important in their dual function of detecting Ca^2+^ and responding through phosphorylation events in opposition to high-temperature signals. There are multiple calcium-dependent protein kinase essentials to react to specific stress stimuli under high-temperature stress. Wang et al. (2018a) also studied the interaction partners of BnCPK2 using bimolecular fluorescence complementation and the split ubiquitin-based pairing system (mbSUS) and revealed a role for BnCPK2 in regulating cell death and modulating ABA signaling and ROS homeostasis, and obtained probable interactions with the NADPH oxidase-like respiratory burst oxidase homolog D (RbohD) (Asano et al., 2012; Wang et al., 2018b). Under heat stress, GmTCTP and GmCDPKSK5 were reported in soybean, and their interaction works in response to heat stress in developing soybean seed (Wang et al., 2017). The burst of cytosolic Ca^2+^ or CDPK stimulates respiratory burst oxidase homolog D (RBOHD), another plasma membrane-located protein with a role in the hydrogen peroxide generation through NADPH oxidase phosphorylation. The downstream signal path of RBOHD is involved in heat shock responses which consist of specific mitogen-activated protein kinases (MAPKs), HSFs, and MBF1c.

### G Protein-Coupled Receptors

G protein-coupled receptors act as plasma membrane-localized receptors in plants that perceive different stress signals and play an essential role in the response of plants under abiotic stresses (Choudhury et al., 2011). These receptors bind to other ligands to sense and transmit the information related to extracellular stress stimuli. Ligand binding to G protein-coupled receptors causes conformational deviations and facilitates the exchange of GTP for GDP, leading to the activation of heterotrimeric guanine nucleotide-binding proteins (G proteins). The association of GPCRs and ligand-bound G proteins activates small Ras-related GTP-binding proteins in canola, which subsequently sets in motion a Ca^2+^ inositol triphosphate (IP3)-mediated signaling pathway under abiotic stress (Shokri-Gharelo and Noparvar, 2018; Nongpiur et al., 2019). Gao et al. (2010) examined *B. napus* and revealed the role of BnGB1 in signal pathways and could also improve the defense system of plants under environmental stresses (Gao et al., 2010).

### Mitogen-Activated Protein Kinase Signaling Cascade

The mitogen-activated protein kinase signaling cascade assimilates and channels signal transduction to express the stress-responsive genes facilitated through phosphorylation and acts as and is involved in converging points in the mechanism of abiotic stress tolerance (Chinnusamy et al., 2004). Mitogen-activated protein kinase signaling cascades are comprised of MAPK kinases (MAP2Ks, MAPKKs, MEKs, and MKKs), MAPKK kinases (MAP3K, MAPKKKs, and MEKK), and MAPKs (MPKs). Mitogen-activated protein kinases function as on-off signaling switches aiming at downstream targets through phosphorylation. Consecutive phosphorylation and de-phosphorylation of threonine or serine residues by MAPKKKs command the activation of MKKs and then tyrosine and threonine residues to activate MPKs (Sun et al., 2014). Then, the activated terminal MAPKs ensue forward with the signal transduction by phosphorylation-arbitrated control of transcription enzymes or factors. However, Liang et al. (2013) identified 12 MPK and 7 MKK members. Sun et al. (2014) identified 66 MAPKKK genes in B. napus. The expressed BnMAPKKK genes were regulated by high-temperature stress and hormone-induced stress stimuli by transmitting external signals to the nucleus via sequential phosphorylation. Mitogen-activated protein kinases act as a signaling molecule sensing and modulating terminal heat stress, which subsequently controls the plant response to heat stress (Krysan and Colcombet, 2018).

### The Pyrabactin Resistance 1-Like Protein

The pyrabactin resistance 1-like protein (BnPYL1-2, BnPYR1-3, and BnPYL7-2) is an essential regulatory constituent of abscisic acid signaling networks in *B. napus* (Di et al., 2018). Abscisic acid is sensed by the ABA receptor (pyrabactin resistance 1-like) in the ABA core signal transduction pathway (Ma et al., 2009; Miyazono et al., 2009). When PYR/PYL is bound by ABA, they inhibit the enzymatic activity of protein phosphatase 2C (PP2C), leading to the release of serine/threonine-protein kinase SRK2 (SnRK2) (Ma et al., 2009). Serine/threonine-protein kinase SRK2 is activated through the activation of loop auto-phosphorylation (Soon et al., 2012), and started by phosphorylate transcription factors, like the abscisic acid-responsive element binding factor (ABF), which is essential to activate ABFs (Kobayashi et al., 2005). These activated abscisic acid-responsive element binding factors enter the nucleus to upregulate the expression of downstream abscisic acid-induced stress-associated genes.

### Matrix Metalloproteinases

Matrix metalloproteinases were found in humans and are a family of zinc-dependent endopeptidases, but a number of matrix metalloproteinases are also located in plants. Speculated results showed that plant matrix metalloproteinases played a role in the growth and development of plants and their response to different stresses. Still, there is a dire need to explore their biological functions (Ratnaparkhe et al., 2009). Pak et al. (1997) revealed the first high plant matrix metalloproteinases (*Gm1-MMP*) which were found to play a significant role in the expansion of soybean leaf. Heat stress-responsive matrix metalloproteinases (*Gm2-MMP*) confer heat stress tolerance by regulating the growth and development of plants which may help researchers to understand the biological functions of the matrix metalloproteinases family in plants (Liu et al., 2017, 2018b).

### Phytochrome A and Phytochrome B

Phytochromes A and B are the most abundant phytochromes in de-etiolated and dark-grown seedlings. PhyB is present in two alternative isoforms: the active Pfr, with a maximum absorbance in the far-red region, and the inactive Pr, which absorbs maximally at the red region (Sakamoto and Kimura, 2018). Phytochrome B mediates signaling pathways to improve plant resistance to environmental stresses by reducing transpiration rate, improving the antioxidant defense system, expressing genes related to plant stress acclimation, and protecting pigments (Junior et al., 2021).

### Heat Shock Factors and Heat Shock Proteins

Heat stress activates all the plasma membrane sensors and generates signals from different transcriptional regulators of HSR (heat shock response) (RbohD, MBF1c, and HSRs) through different kinases. The chloroplast is projected as a heat sensor as its translation ability of proteins triggers retrograde signals to heat receptive genes, which are HsfA2-dependent themselves (Liu et al., 2015). HSFs (heat shock transcription factors) are activated by calmodulin, Hsp90, and mitogen-activated protein kinases. Signal transduction includes various phases like activation of HSFs and their expression, which leads to the onset of thermo-tolerance (Saidi et al., 2011).

A prominent event is heat-tolerance acquisition, transcription, and translation of heat shock factors (HSFs) and heat shock proteins (HSPs). Consequently, the constitutive over-expression of these genes and proteins is well-established to enhance heat tolerance (Vierling, 1991). Heat shock factors (HSFs) and dehydration responsive element-binding (DREB) protein families were also identified in *Brassica juncea* (Bhardwaj et al., 2015). The promoter regions of the soybean HSFs contained cis-elements that likely participate in drought, low temperature, and ABA stress responses. GmHsp90A2, GmRAR1, GmSGT1, GmSBH1P, and GmSBH1 are essential chaperones of the protective stress response in soybean (Chen et al., 2019; Huang et al., 2019b), while LusHSF responds in linseed (Saha et al., 2019). These regulators play their role in making the interaction between the MBF1c ethylene activated pathway and HSP allied signaling. This coordination and members of the DREB family facilitate the responses against heat stress. The plant's highly conserved heat stress response has four putative sensors that initiate the heat stress response (Mittler et al., 2012). GmHsp90A2 *was* identified as a positive regulator under heat stress in soybean, which interacted with GmHsp90A1 and exhibited increased tolerance to heat stress through higher chlorophyll and lower malondialdehyde (MDA) contents in plants (Huang et al., 2019b). High temperature can significantly affect gene expression during flowering as thermo-sensitive genic male sterility (TGMS) provides an adequate foundation for male fertility research in *B. napus*. We also found that transcription factor box transcription factor (MADS), Nuclear transcription factor Y (NFY), heat shock transcription factor (HSF), MYB/C, and WRKY might play a crucial role in male fertility under the high-temperature condition (Gao et al., 2021). *BnaMYBs* improve tolerance to cold, heat, drought, and salinity by regulating ROS defense genes (Chen et al., 2016; Hajiebrahimi et al., 2017). Demirel et al. (2014) studied 25 ESTs (express sequence tags), out of which 16 were homologous to known genes. The genes, namely RPS14, CTL2, LSm8, ABCC3, and CIPK, were downregulated, while FPGS, TH1, GhHS128, GhHS126, and IAR3 were upregulated, but expressions of psaB-rps14 and PP2C were not altered, owing to short-term heat stress in cotton. Hence these putative sensors activate heat stress-responsive genes to enhance thermo-tolerance, but the hierarchical order and relation between these pathways remain unclear.

**Figure 1:**
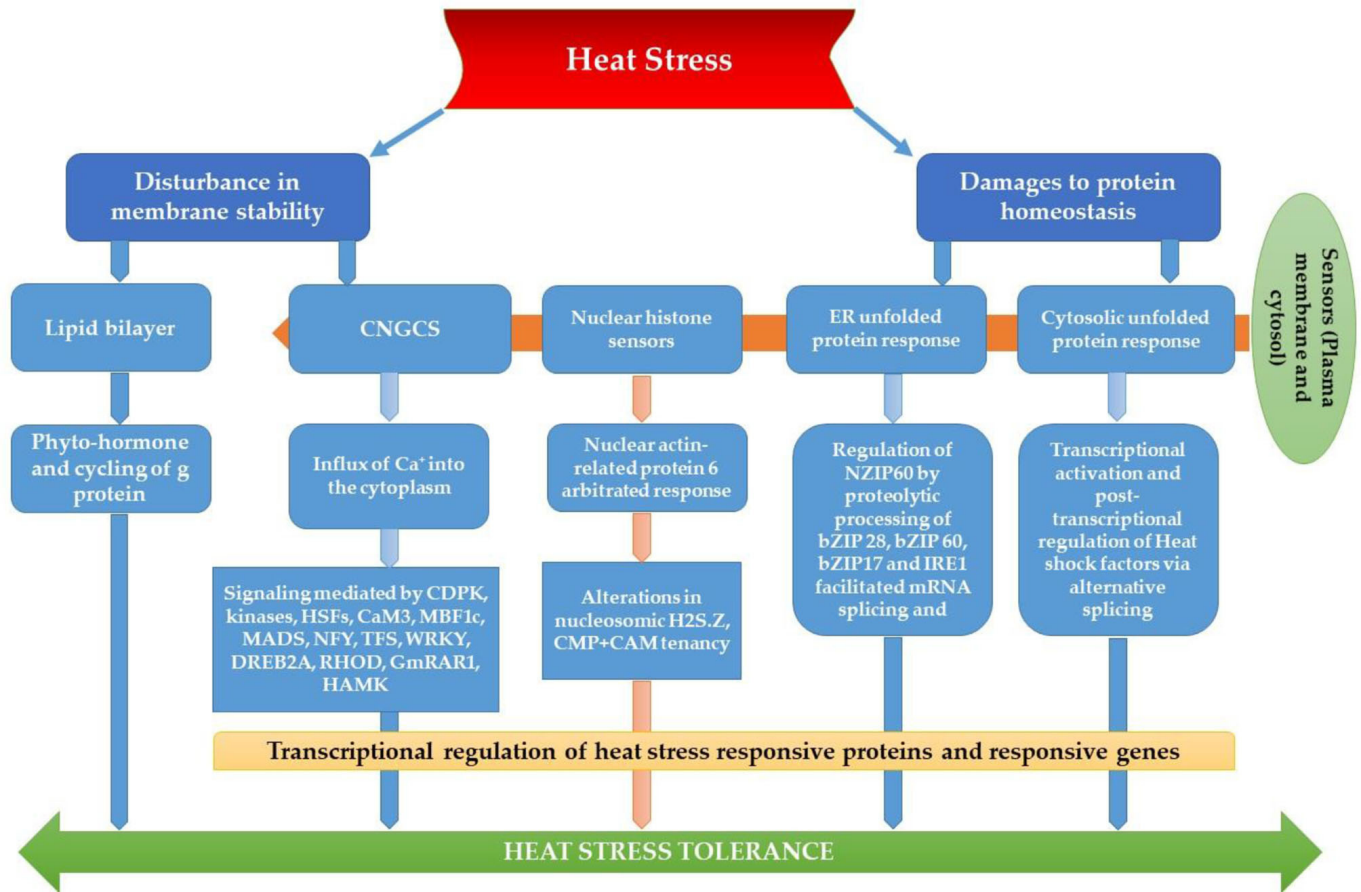
Plant thermo-sensors and main signal transduction pathways implicated in heat stress response and thermo-tolerance (modified from Bokszczanin and Fragkostefanakis, 2013).

**Figure 2:**
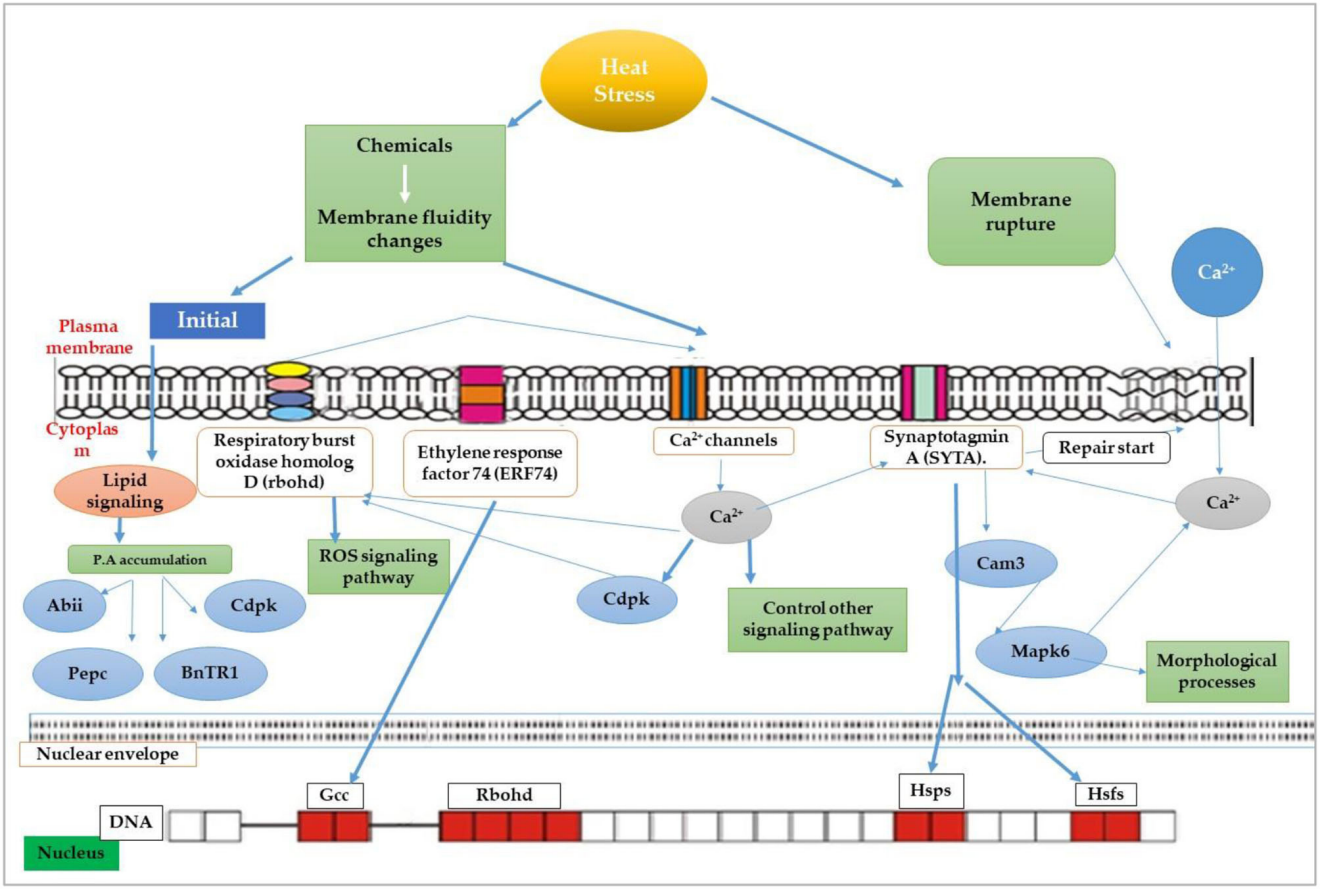
Membrane heat sensors and signal transduction pathways through various receptors across the plasma membrane.

The most recognized putative heat sensors in the plasma membrane are Ca^2+^ channels known as cyclic nucleotide gated calcium channels (CNGCS), a nucleosome containing histone variant (H2A.Z), and unfolded protein sensors; (a) ER-UPR and (b) Cyt-UPR as depicted in Figure 1. In a calcium signaling pathway, calcium interacts with the number of signaling molecules inside the cell to trigger the heat stress response. To operate HSPs expressions, Ca^2+^ interacts with HSFs (heat stress transcription factors), *via* CBK (Ca^2+^/CaM3 binding protein kinases) and CaM3 (calmodulin 3). Ca^2+^ is required for the activity of RbohD against ROS stress (respiratory burst oxidase homolog D) or CDPKs (calcium-dependent protein kinases). It can repair the membrane with synaptotagmin A (SYTA) (Sajid et al., 2018). Calcium-dependent protein kinases (CDPKs), identified only in plants, are a vital regulatory protein decoding calcium signals activated by various environmental stimuli. However, only CDPK 8 from the CDPK family has been reported to have a role in abiotic stress response via scavenging H_2_O_2_ by catalase-3 (Zou et al., 2015). The expressions of other HSPs and HSFs initiated by Ca^2+^ regulate master HSPs and HSFs and trigger the enzyme activity to prepare the plant for heat tress tolerance, as shown in Figure 2. An alternative complementing heat-sensing mechanism proposes that the primary temperature sensor of the cell is located in the plasma membrane and that Ca^2+^ permeable channels act as the earliest temperature-sensing component of the plant heat stress response (Saidi et al., 2009). Heat stress activates cell sensors, and among those, plasma membrane sensors activate calcium channels which causes the inward flux of calcium.

## Heat Stress at Germination and Vegetative Stage

**Figure 3:**
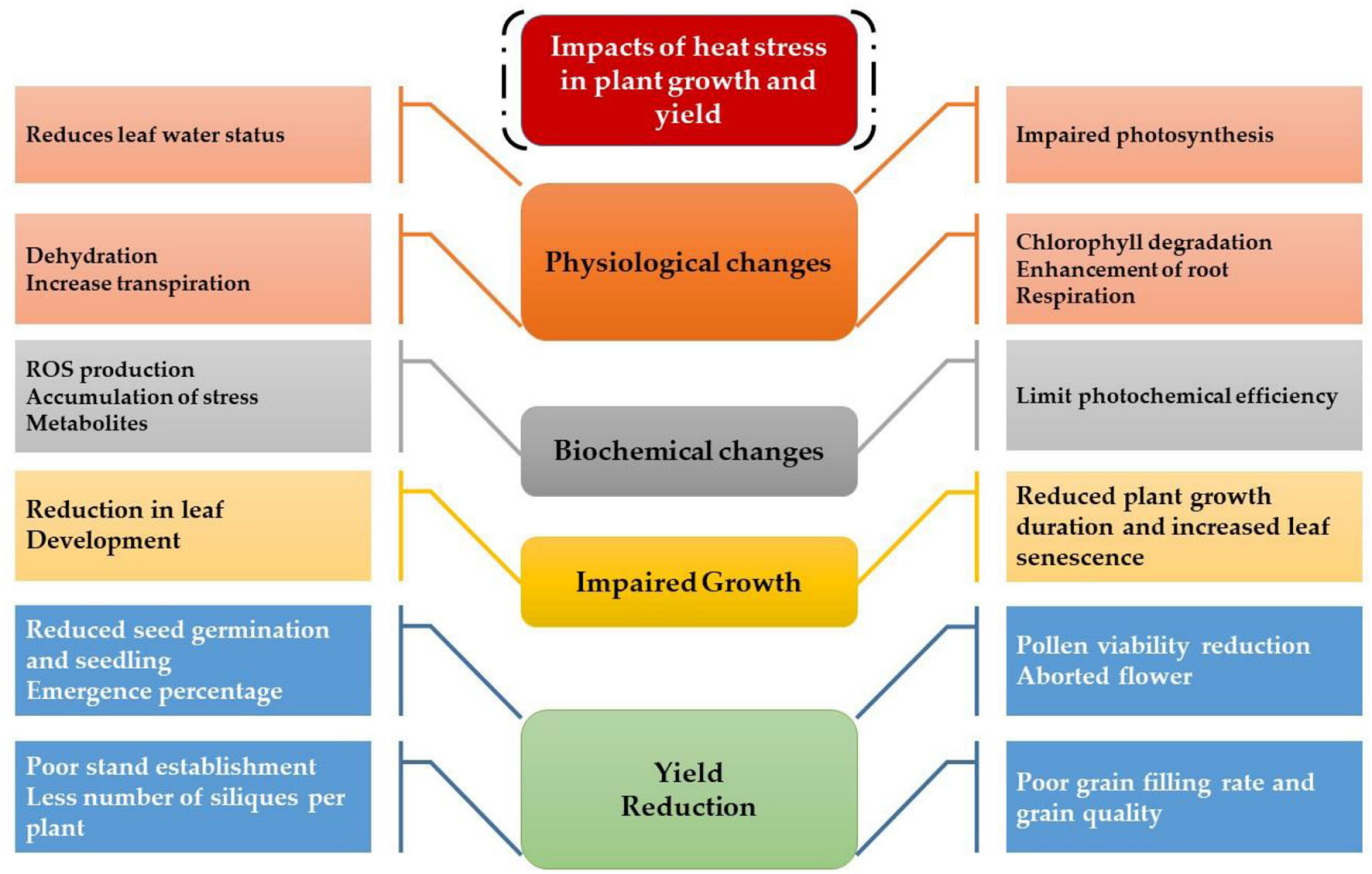
Impact of heat stress on physiological, biochemical, growth, and yield responses in plants.

The germination and emergence ratio of a crop is the critical phenomenon to get the optimum planting density and crop performance in the field. High temperature resulted in poor germination and poor stand establishment in the Indian Brassica germplasm (Azharudheen et al., 2013). Recently, induction of varying degrees of secondary dormancy at sub and supra-optimal temperature regimes were detected among rapeseed cultivars (Gorzin et al., 2020). Heat stress damages plant morphology and is manifested by symptoms on vegetative parts such as leaf sunburn, scorching effects of heat on leaves, twigs, buds, branches, stems, and fruits, reduction in root to shoot ratio, affects plant meristems, and leaf senescence (De la Haba et al., 2014) with subsequent abscission and ultimate reduction in seed yield (Bita and Gerats, 2013). As temperature increases, the plant development builds up to a certain extent and decreases afterward (Wahid et al., 2007). The impaired growth and development symptoms were observed in Brassica (Angadi et al., 2000), soybean (Piramila et al., 2012), and linseed (Gusta et al., 1997) under high-temperature stress. Ahmad et al. (2021a) reported that high temperature (35°C) during anthesis reduces chlorophyll content, photosynthetic rate, and leaf water status in camellia and canola genotypes, leading to reduced plant growth and seed yield. Canola growth was negatively affected above 28°C by reducing plant height, root length, and biomass accumulation due to impaired photosynthetic rate and stomatal conductance (Waraich et al., 2021b). The consequences of heat stress for plant growth and development are presented in Figure 3.

## Heat Stress at Reproductive Stage

All the plant growth stages could be poorly affected by thermal stress, but the biggest concern of the agricultural world is the reproductive phase. The central part of the world's food supply comes from the flowering plant through sexual reproduction. The first few hours of the reproductive phase are important in fertilization, as a small spell of heat stress occurs, which can be fatal to the whole process (Xi, 1991). Similarly, the reproductive stage is considered the most sensitive stage to be affected by heat stress in Brassica (Young et al., 2004; Ihsan et al., 2019). The disruption of the plant's metabolic functions due to high temperature is associated with a consequent reduction in light interception due to a shortening of the growth phases in terms of both size and time. It also has an adverse effect on carbon assimilation, leading to the formation of small and deformed organelles (Maestri et al., 2002). A very fatal heat stress response has been observed in male and female reproductive parts, which impairs pollen viability and germination, inhibits pollen tube growth, impairs receptivity and function of the stigma and ovary, causes fertilization arrest, inhibits embryogenesis, impairs egg viability, and induces ovarian abortion and poor seed set. Brassica plants have shown a poor seed set when exposed to heat stress (Angadi et al., 2000; Morrison et al., 2016). It has been observed that late flowering to early seed setting is the most susceptible growth stage to heat stress in groundnut (*A. hypogaea*) (Prasad et al., 1999). One more example of this occurs in cotton. In this case, the most heat-sensitive stages in cotton are pollen and pollen tube development and fertilization in reproductive growth. High-temperature stress reduces the number of sympodial and monopodial branches, number of bolls, seeds per boll and their weight, and the boll development process (Ekinci et al., 2017; Rahman et al., 2019). The canola grain yield drastically reduced when exposed to high night temperatures during the reproductive stage (Pokharel et al., 2020; Chen et al., 2021b).

## Oil Quantity and Quality

Among the plant reserves, oils are the most energetic reserves, providing humans with many essential fatty acids and calories that must be part of the daily diet. It is synthesized in plastids, oil bodies, and triacylglycerol (TAG) molecules that accumulate outside the plastids in the endoplasmic reticulum (ER). Exposure to heat episodes has detrimental effects on cell organelles (plastids, ER, and oil bodies), it also induces the denaturation of enzymes, which could lead to the impairment of the mechanism of oil synthesis (Haung et al., 2019). Although under heat stress conditions, the full mechanism of oil accumulation and photosynthesis in *B. napus* remains unclear, it is known that under these conditions, the sugar content increases because seed oil accumulation is reduced, leading to impaired carbohydrate incorporation into TAG (Haung et al., 2019). Heat stress lessens the role of a number of sugar transporter genes, resulting in the imperfect incorporation of carbohydrates into triacylglycerol's units. Taken together, the results confirmed that perturbations in the mechanism of seed photosynthesis, impaired integration of carbohydrates into triacylglycerol, and transcriptional deregulation of the BnWRI1 pathway due to heat stress are the main reasons for less oil accumulation (Haung et al., 2019). The relationship of oil concentration with temperature is linear. As temperature increases, the concentration of oleic acid increases linearly, and at the same time, linoleic acid decreases linearly (Thomas et al., 2003; Lanna et al., 2005).

Additionally, linoleic and linolenic acids, isoflavones content, and iodine number also decreased. All these factors added to reducing oil content in soybean seeds (Lanna et al., 2005). The oil yield showed a linear correlation to thousand seed weight, pod length, and seeds per pod of the Brassica species accessions in tropical environments, especially under high-temperature regimes indicating a promising potential as alternative oilseed crops for biodiesel production in tropical conditions (Bassegio and Zanotto, 2020).

## Physiological and Metabolic Basis for Reproductive Failure Under Heat Stress

The vulnerability of plants to heat stress varies with the different growth stages. At the same time, the reproductive phase is also susceptible due to its sensitive organelles that surrender to heat changes. Heat stress reduces the plant's photosynthetic capacity, resulting in a lack of resources for the reproduction process in the genotypic and reproductive tissues (Ahmad et al., 2021a). Some causes of reproductive failure and male sterility in plants are related to the genes responsible for the tapetum and pollen functioning, which are altered by heat stress occasioning their degradation. Carbohydrate metabolic enzymes, including sucrose synthase, vacuolar inverses, and sugar transporters, are influenced by heat stress reducing the pollen viability (Zandalinas et al., 2018). The accumulation of soluble carbohydrates in pollen is reduced by low sucrose-starch turnover due to downregulation of the enzymes sucrose synthase and invertase (Hedhly, 2011). Under heat stress, cell proliferation arrest produces changes in chloroplast development, abnormalities in mitochondria, and distended vacuoles (Sakata et al., 2010; Wani and Kumar, 2020). In stigmatic tissues and pollen grains, carbohydrate accumulation is disrupted due to changes in the partitioning of assimilates between the apoplast and symplastic phloem filling, which impairs pollen grain viability. High-temperature stress leads to inhibition of starch production in oilseeds (Thuzar et al., 2010), associated with seed setting and oil accumulation under heat stress. The drastic reduction in grain weight was directly linked with electrolyte leakage and membrane damage resulting in low seed yield under terminal heat stress in *B. juncea* (Kavita and Pandey, 2017).

## Physiological Responses

**Figure 4:**
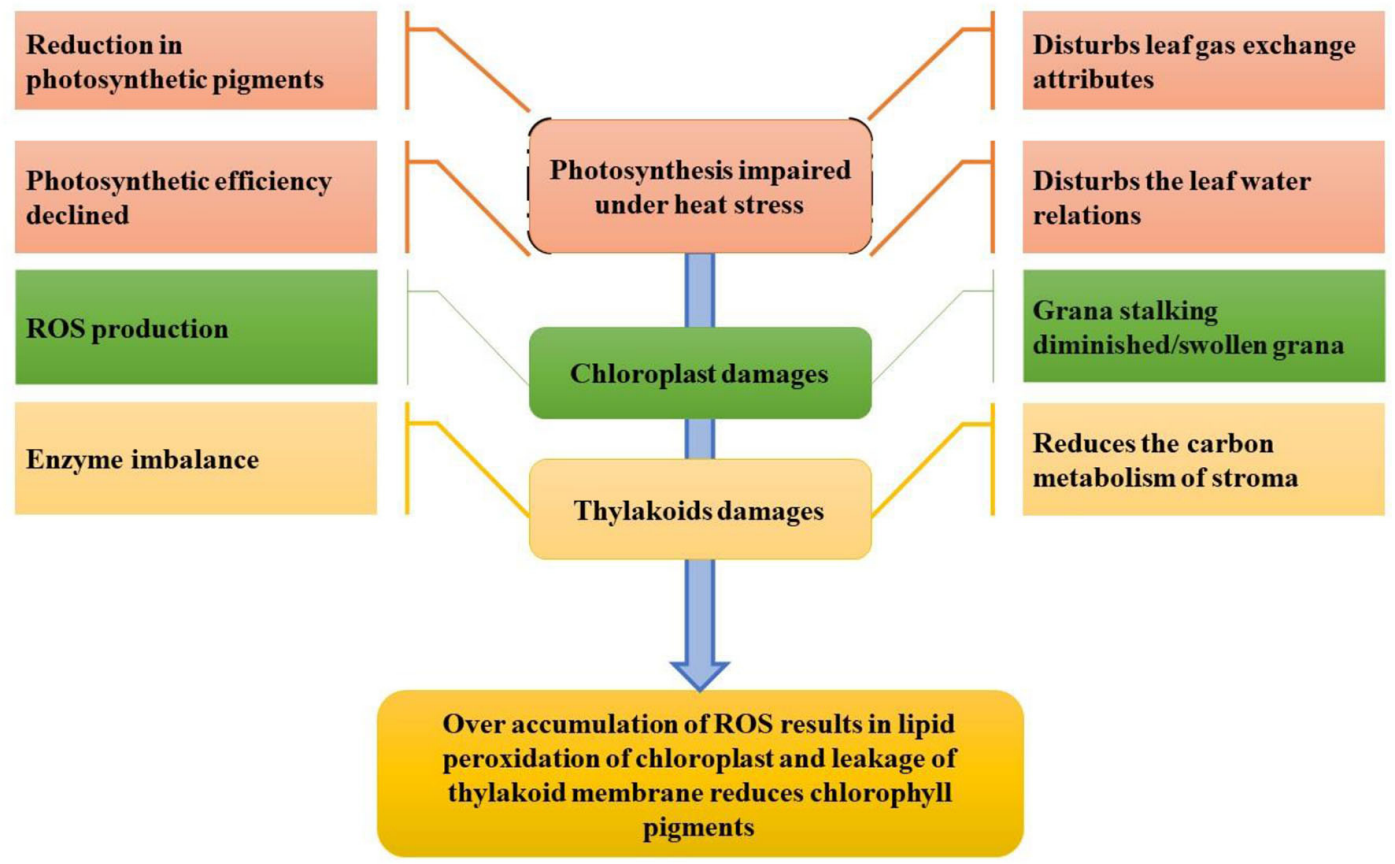
Impact of heat stress on photosynthesis and the photosynthetic system (conceived from Nadeem et al., 2018).

Initially, heat stress damages the chloroplast proteins complex and inhibits the enzymatic activity (Ahmad et al., 2010; Hasanuzzaman et al., 2020a). Under high-temperature environments, the chloroplasts are unfolded and vulnerable to rapid degradation of chloroplast proteins (Dutta et al., 2009), particularly one of the significant core subunits in photosystem II (PSII), protein D1, is the most vulnerable to heat stress damage. The impact of heat stress at the cellular level is devastating because it damages membrane stability, inactivates enzymes in chloroplasts and mitochondria, also promotes protein and enzyme degradation, decreases carbohydrate and protein synthesis, reduces carbon metabolism, and alters microtubule organization by expanding and elongating cells, ultimately damaging the cytoskeleton (Bita and Gerats, 2013). Heat stress affects all the physiological processes, but the most sensitive among all is photosynthesis (Crafts-Brandner and Salvucci, 2002; Hasanuzzaman et al., 2020b) as shown in Figure 4. The effect of elevated temperatures on photosynthesis can be seen in several instances. Heat disrupts the integrity of the thylakoid membrane and damages photosystems I and II as well as the oxygen complex, affecting phosphorylation (Rexroth et al., 2011). PSII is the most sensitive photosystem (Bibi et al., 2008); thus, severe thermal damage to PSII results in disrupting electron transport and ATP synthesis during the photosynthetic process (Wang et al., 2018a).

ROS production has detrimental effects on photosynthetic machinery and PSII (Bita and Gerats, 2013; Elferjani and Soolanayakanahally, 2018). Leaf chlorophyll contents have a negative relation with heat stress resulting in less photosynthesis at 38/32°C that lowers the chlorophyll content leading to a decrease in the sucrose content. In addition, it suppresses the process of carbon fixation in photosynthesis by reducing chlorophyll content (Liu and Hang, 2000; Ahmad et al., 2021a), reducing the quantum yield of photosystem II (Bibi et al., 2008), reducing e-transport (Wise et al., 2004) due to leakage in the thylakoid membrane, inactivating rubisco activation, and increasing cyclic photophosphorylation. Heat stress affects the plant in many ways. For example, excess water can also be siphoned off due to increased transpiration, leading to reduced plant turgidity and disruption of physiological processes (Tsukaguchi et al., 2003). Heat stress affects water relations in the plant by affecting osmotic adjustment due to the poor photosynthetic capacity of the plant (PSII is the most sensitive part), reducing sugar content, decreasing the osmotic potential of leaves, and increasing transpiration rate (Hemantaranjan et al., 2018).

## Heat Stress and Phytohormones/Signaling Molecules

The plant faces several external and internal stimuli during its lifespan. Therefore, they need to regulate their growth and development in reaction to these stimuli (Li et al., 2021a). A small group of signaling molecules known as phytohormones (abscisic acid, brassinosteroids, cytokinin, salicylic acid, jasmonate, and ethylene) present in small quantities in the cell and help to mediate the response to stimuli. Although ABA is the primary regulator of the response to abiotic stress among phytohormones, increasing evidence points to the involvement of other phytohormones. The nature of phytohormone-mediated regulation of heat stress tolerance is a complex phenomenon, as they can act either directly respond or orchestrate the response to high-temperature stress by engaging other phytohormones, including reactive oxygen species, MAP kinases, soluble sugars, and secondary messengers through crosstalk networks (Smékalová et al., 2014; Ljung et al., 2015). The role of phytohormone-induced regulation of stress tolerance has been extensively reviewed (Peleg and Blumwald, 2011; Balfagón et al., 2019). These phytohormones play a crucial role in acclimatizing plants to rapidly changing environmental conditions by regulating transitions between sources and sinks, growth and development, and well-known nutrient distribution (Nazar et al., 2017). Plant hormones mediate soybean plant tolerance to high-temperature stress by enhancing plant growth and development through regulation of the antioxidant defense system, interaction with plant hormones, and reorganization of biochemical metabolism (Imran et al., 2021).

**Figure 5:**
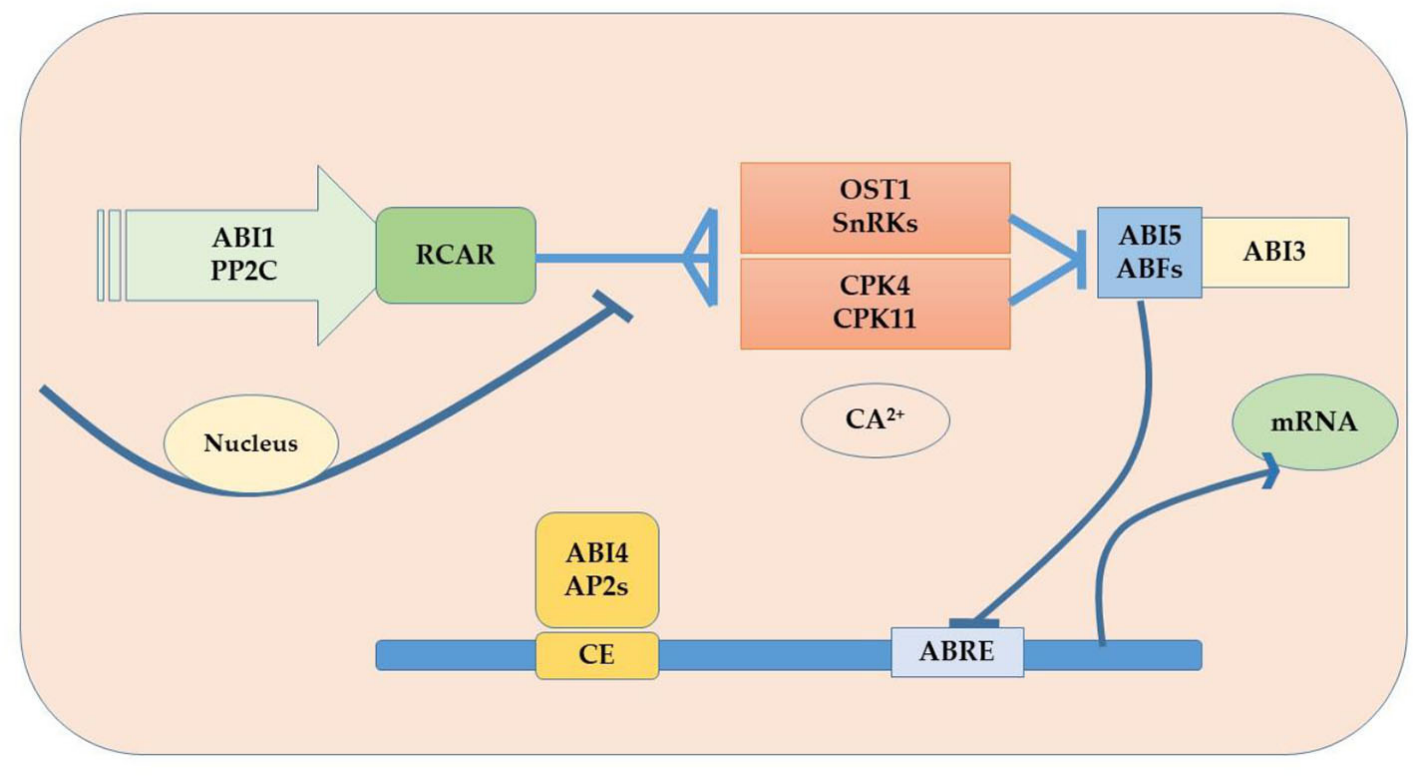
ABA signaling pathway in oilseed crops.

**Figure 6:**
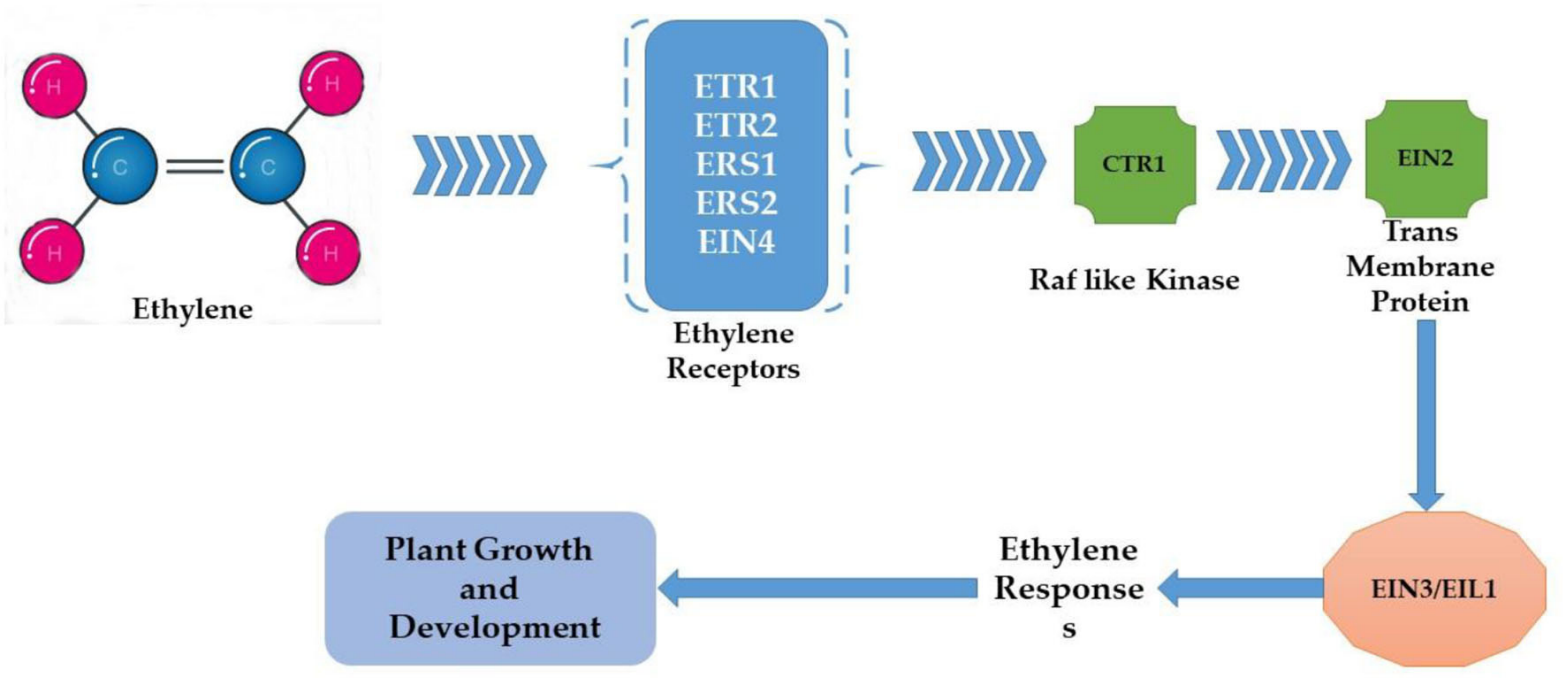
Ethylene signaling pathway under heat stress.

Plants are sessile organisms and their survival in elevated temperature depends upon various factors. One of the most vital substances used in response to external stimuli is endogenously produced phytohormones, which regulate different molecular and physiological reactions (Li et al., 2021b). Phytohormones might act where they were synthesized or can be transported wherever needed (Peleg and Blumwald, 2011). Some of the critical roles phytohormones play to face abiotic stress tolerance include, for example, the increment in the synthesis of cytokinins (CKs) under water stress conditions for better functioning. Melatonin supplementation regulates the plant defense system by improving the activity of antioxidants (superoxide dismutase, ascorbate peroxidase, peroxidase, and catalase) and their genes (GmPOD1, GmSOD, GmAPX, and GmCAT1), biochemicals (phenolic substances, flavonoids, and proline), and polyamines (spermine, spermidine, and putrescine), and also by downregulating stress hormone biosynthesis including abscisic acid content, downregulated gmNCED3 (abscisic acid biosynthesis gene), and upregulated catabolic genes (CYP707A1 and CYP707A2) in soybean. In addition, melatonin induced the expression of heat shock transcription factor (gmHsfA2), heat shock protein 90 (gmHsp90), and indicated detoxification of reactive oxygen species through the H_2_O_2_-mediated signaling pathway (Imran et al., 2021). Kazan and Manners (2013) delineates the evolutionary role that a second phytohormone auxin (IAA) plays in stress tolerance because of its biosynthesis, signaling, and transportation apparatus present in the cell; some other studies found evidence of the role of gibberellins (GAs) to ameliorate adverse circumstances, for instance Colebrook et al. (2014) found that gibberellins role in abiotic stress tolerance has been increasing with time; another phytohormone that takes part in the response to abiotic stress is abscisic acid (ABA), the level of ABA upsurges in plants under unfavorable conditions modifying gene expression and activating signaling pathways (O'Brien and Benková, 2013) as shown in Figure 5. Ethylene (ET) also plays an important role in the acclimatization in adverse conditions (Gamalero and Glick, 2012) and also modulates gene expressions as shown in Figure 6 (Klay et al., 2014). Jasmonates (JAs), strigolactone (SL), and salicylic acid (SA) govern growth and development and fruit ripening in abiotic stresses (Rivas-San Vicente and Plasencia, 2011); jasmonate (JAs) regulates plant defense to stressful conditions, and brassinosteroids (BRs) are an essential phytohormone that have a role in the heat stress tolerance in plants (Bajguz, 2011; Janeczko et al., 2011). Endogenous abscisic acid concentration was significantly elevated by heat stress (45°C) alone and doubled by heat stress plus brassinosteroids. These results suggested that the well-known enhancement of heat stress tolerance was obtained due to brassinosteroid-induced elevation in endogenous abscisic acid concentration (Kurepin et al., 2008). Jasmonate is required to regulate specific transcriptional responses unique to the heat and high light stress combinations in the chloroplast, especially D1 protein in PSII (Balfagón et al., 2019). All these are key players to provide developmental plasticity in plant growth. The role of phytohormones in oilseed crops also extraordinarily starts at the biosynthesis of oil content using a signal transduction mechanism and has a role in the performance of many growth and developmental processes. It is well-documented that exogenous application of phytohormones mitigates the negative effects of heat stress in canola (Kurepin et al., 2008). Similarly, supplementation with brassinolide at the seedling stage can enhance thermo-tolerance by increasing endogenous ABA levels. However, treatment with 24 epibrassinolides also increases heat stress tolerance (Kagale et al., 2007). qRT-PCR analysis showed that the expression levels of gibberellin biosynthesis pathway genes (GmGA3ox1, GmGA3ox2, and GmGA3) and auxin biosynthesis pathway genes (GmYUCCA3, GmYUCCA5, and GmYUCCA7) significantly increased upon interaction with high temperature and supplementation of gibberellins and auxin, which improved the performance of soybean plants by improving hypocotyl elongation under high-temperature stress (Bawa et al., 2020).

## Mechanism of Heat Stress Tolerance in Plants

The global climate is changing due to various anthropogenic factors that influence temperature regimes (Ahmad et al., 2020). There are several mechanisms, including phenological, physiological, morphological, and biochemical mechanisms, that plants exhibit for their survival under high-temperature conditions (Ghaffar et al., 2020) since plants are sessile in nature, which limits them to a specific range of responses to external stimuli that vary at different stages of growth and have flexible relevance to physiological and cellular mechanisms of protection and acclimatization (Ahmad et al., 2021a). The prime stress indications (e.g., variations in temperature, ionic effects, osmotic effects, membrane uncertainty) would activate the signaling and transcript control that triggers the mechanism of stress responses to restore the homeostasis and repair the plasma membrane. Bohnert et al. (2006) examined cell death due to devastation in the structural and functional proteins and irreversible damage in the homeostasis of the cell because of an insufficient response at different steps of signaling and gene expression processes. Understanding the various mechanisms of the reaction of plants to stress and their importance in the acquisition of thermo-tolerance is of great importance. Under heat stress, plants activate a variety of mechanisms, including accumulation of metabolites (HSPs, osmoprotectants, antioxidants), ion carriers, late embryo abundant proteins, free radical scavengers, transcriptional control, and factors involved in signaling, which are fundamentally very important for stress alleviation (Bokszczanin and Fragkostefanakis, 2013). By observing the heat, signaling, and metabolite production that help the plant survive adverse conditions, a chain of mechanisms and variations began. The impacts of heat stress are evident at different stages and in aspects such as plasma membrane fluidity, biochemical mechanisms in cytoplasmic organelles, and cytosol (Sung et al., 2003). The primary sight of damage resulting from heat stress is plasma-lemma which results in damage to the lipid bilayer plasma membrane. This implies the initiation of cytoskeleton reorganization and Ca2+ influx, leading to the regulation of CDPK and MAPK.

Heat stress results in the production of ROS in various organelles (peroxisomes, chloroplasts, and mitochondria), which are important in the signaling mechanism, activation of antioxidant enzymes, HSPs, and restoring the balance of osmolyte concentration that maintains the water balance of the cell (Bohnert et al., 2006). Plants can adopt several stress mechanisms, with the ability being associated with acquiring thermo-tolerance (Maestri et al., 2002). During conditions of heat stress, HSP chaperones play essential roles in signal transduction and gene expression, as well as in the regulation of cellular redox balance, protection of photosynthesis, protein and membrane repair, osmolyte production (Diamant et al., 2001), and antioxidant production. The response to heat shock can be controlled at the transcriptional and translational levels. The cis-acting DNA sequence, heat shock element (HSE), and LusHSF genes have been found to play an important role in heat-induced transcription (Nover and Baniwal, 2006; Saha et al., 2019). During heat episodes, a protective mechanism is also associated with increased thermo-tolerance of the photosynthetic apparatus (Hemantaranjan et al., 2014). Consequently, the induction of thermo-tolerance for plant protection under such conditions is directly linked to the ability to detoxify and scavenge radical ROS, leading to plant thermostability (Hameed et al., 2012). Although many attempts have been made, Iaccthere is still very little literature on ROS production and scattering. The saturation of membrane lipids under heat stress tolerance increases the content of trans-3-hexadecanoic acid (among phospholipids) and linolenic acid (among galactolipids). However, it is still unclear whether either low membrane lipid saturation or higher membrane lipid saturation is beneficial in mitigating heat stress (Klueva et al., 2001). Total soluble proteins play a vital role in improving heat stress tolerance in oilseed crops, including camellia and oilseed rape by improving plant water relations and gas exchange properties that help improve vegetative and reproductive growth under high heat stress (Ahmad et al., 2021b,c). During heat stress, the photosynthetic electron transport chain is protected by the localization of LMW-HSPs with the chloroplast membrane (Heckathorn et al., 1998). Variations in expressions of genes are a vital part of the heat stress tolerance response. Yang et al. (2006) observed a rapid shift of gene expression under heat stress in inhibiting HSP complement expression. The splicing of many mRNAs could be restrained by heat stress. Unfavorable high-temperature heat stress conditions may also destabilize the non-heat stress-induced proteins encoded by mRNAs. Investigations show that the presence of introns in the HSPs is why the mRNAs were sliced properly compared to proteins with no introns. Accordingly, a number of genes were identified to confer thermo-tolerance in plants, i.e., regulated glutaredoxin, ascorbate peroxidase, heat shock factors, heat shock proteins, and downregulated FAD3-2 and FAD7 to improve resistance in plants against heat shocks induced by high-temperature stress (Murakami et al., 2000; Lwe et al., 2020). However, further studies are needed to elucidate the mechanism of professional transcriptional alteration and transformations of HSP-encoding mRNA under lethal high temperatures. Although, Ca^2+^ acts as a regulator of many physiological and biochemical processes in response to high-temperature stress in plants (Yang et al., 2013), transient elevation of free Ca^2+^ in the cytoplast can be detected in plants in response to various stresses, such as high temperatures. The fact that Ca^2+^ improves plant resistance has been linked to the maintenance of higher photosynthetic rates under stress, histone sensors, and unfolded protein response sensors in the endoplasmic reticulum (ER), RBOHD, plasma membrane channels (which transiently open and induce Ca^2+^ entry flux into the cytosol), phytochrome B (Mittler et al., 2012; Vu et al., 2019), PSII reaction center stability, ROS detoxification, and high light-induced Ca^2+^ influx into chloroplasts, which regulates antioxidant processes to mitigate high temperature-induced oxidative stress (Yang et al., 2013; Gilroy et al., 2016). For example, the superoxide anion, the initial product of photoreduction of O_2_, is dismuted by superoxide dismutase to H_2_O_2_ and O_2_ (Noctor and Foyer, 1998). H_2_O_2_ is then converted into water by ascorbate peroxidase (APX). Furthermore, exogenous Ca^2+^ could improve the non-photochemical quenching of chlorophyll fluorescence (Ai et al., 2007), protecting the photosynthetic machinery from inactivation and damage caused by excess irradiance (Horton et al., 1996). In addition, a class of HSF family TFs (e.g., HSFA1s) and the Ca^2+^ /CaM signal transduction pathway regulate plant responses to high temperatures (Yang et al., 2013; Cortijo et al., 2017; Ohama et al., 2017). Pretreatment of plants with hydrogen peroxide or phytohormones increases the expression of genes encoding enzymes such as catalase, which scavenges ROS, and redox regulators such as glutaredoxin, which improve plant temperature tolerance (Wang et al., 2014; Devireddy et al., 2021).

### Acquired Thermo-Tolerance

In plants, the capability to survive under lethal high-temperature stress following adaptations with sub-lethal high temperature or the capability of a living thing to survive in a severely high temperature is referred to as acquired thermo-tolerance (Jagadish et al., 2021). Like other organisms, plants cope with severe high temperatures by acquiring thermo-tolerance within a few hours; they also have an inherent characteristic to survive in lethal temperatures (Lin et al., 2014). Stress memory is defined as the process of storage and retrieval of information acquired during initial exposure to stress (Crisp et al., 2016; Hilker and Schmülling, 2019). Naturally, plants face different gradual temperature ranges and acclimatize to these otherwise lethal ranges, which is an independent cellular phenomenon of thermo-tolerance acquisition that results from the pretreatment under high temperature for short periods correlated with a higher activity of antioxidant enzymes (Collado-González et al., 2021). This acquisition of thermo-tolerance is used as a yardstick to evaluate the thermo-tolerant and thermo-sensitive genotypes of oilseeds like groundnut and sunflower (Awais et al., 2017b), and elaborate the functions of different stress genes. Thermo-tolerance acquisition is not a single-step phenomenon; rather, it has different phases. Certain factors affect the acquisition of thermo-tolerance like growth stage, acclimation methods, and crosstalk between acquired thermo-tolerance and stress tolerance. These key factors protect the cells from the detritus impacts of heat stress in acquired thermo-tolerance (Jespersen, 2020). A piece of useful information regarding heat stress effects can be revealed by the inspection of all the hostile effects instigated by extreme heat as the responses of thermal stress in the plant are related to other types of stress (Rahaman et al., 2018). The HSRs, referred to as a transitory restructuring of gene expressions, are a preserved biological response of different organisms and cells to eminent temperatures (Schöffl et al., 1999). The upregulated genes under high-temperature stress encoded many heat shock factor (HSF)-like proteins such as HsfB2A (Bra029292) and heat shock proteins (HSPs), including high molecular weight HSPs. Heat stress also upregulated some components of HSR, including ROS-scavenging genes such as protein kinases, glutathione peroxidase 2 (Bra022853, BrGPX2), and phosphatases. At the same time, CYP707A3 (Bra025083, Bra021965) was involved in membrane leakage, but many transcription factor (TF) genes, including DREB2A (Bra005852), were involved in the acquisition of heat stress tolerance in bryophytes (Dong et al., 2015). HSP is a vital apparatus to examine the molecular mechanism of heat stress tolerance and gene-expression regulation in plants. The total temperature needed for the initiation of HSR accords with the optimum temperature of a specific species, which is 5–10°C more than normal thermic conditions. It involves the education of HSPs and, therefore, a higher level of thermo-tolerance acquisition. In the transitory synthesis of HSPs, results showed that the signal that triggers the reaction is either lost, deactivated, or not documented (Burke, 2001; Lwe et al., 2020). The direct role of HSPs in thermo-tolerance is challenging to determine, but its involvement in acquired heat tolerance is a logical model (Burke, 2001). So, the acquired thermo-tolerance in plants obtained through natural phenomenon prompted by a gradual acquaintance to heat periods or biological synthesis of pertinent compounds, although cost-intensive, is a vital and hypothetically a critical strategy. This mechanism is primarily associated with the display of HSR and acquired by the restructuring of gene expression, letting plants survive under the sub-lethal temperature. Edelman et al. (1988) performed studies in soybean seedlings and revealed that as the temperature reached 40°C, the protein pattern shifted from normal proteins to HSPs to acquire thermo-tolerance. Remarkably, a minimal but significant level of acquired thermo-tolerance can be achieved in plants by inducing the expression of a small number of genes regulated by other transcription factors, such as the NAC069 TF (Wang et al., 2016a), MYB-related genes (FAR1, bZIP, and mTERF) (Zhou et al., 2016), MADS-box, MYB41 (Wu et al., 2018), NAC (Saha et al., 2021), and CWM-related genes (Wang et al., 2016b). Kinases including MAPKKK (mitogen-activated protein kinase) (Sun et al., 2014) and superoxide dismutase (SOD)-related genes (BnSOD) (Su et al., 2021), CBL (calcineurin B-like proteins) and CIPK (CBL-interacting protein kinases) (Yuan et al., 2014), and CPK (calcium-dependent protein kinase) and transporters including SUT/SUC and SWEET (Jian et al., 2016) have been identified and found to provide genetic resources for improving high temperature tolerance traits in *Brassica*.

### Antioxidant Defense in Response to Heat-Induced Oxidative Stress

**Figure 7:**
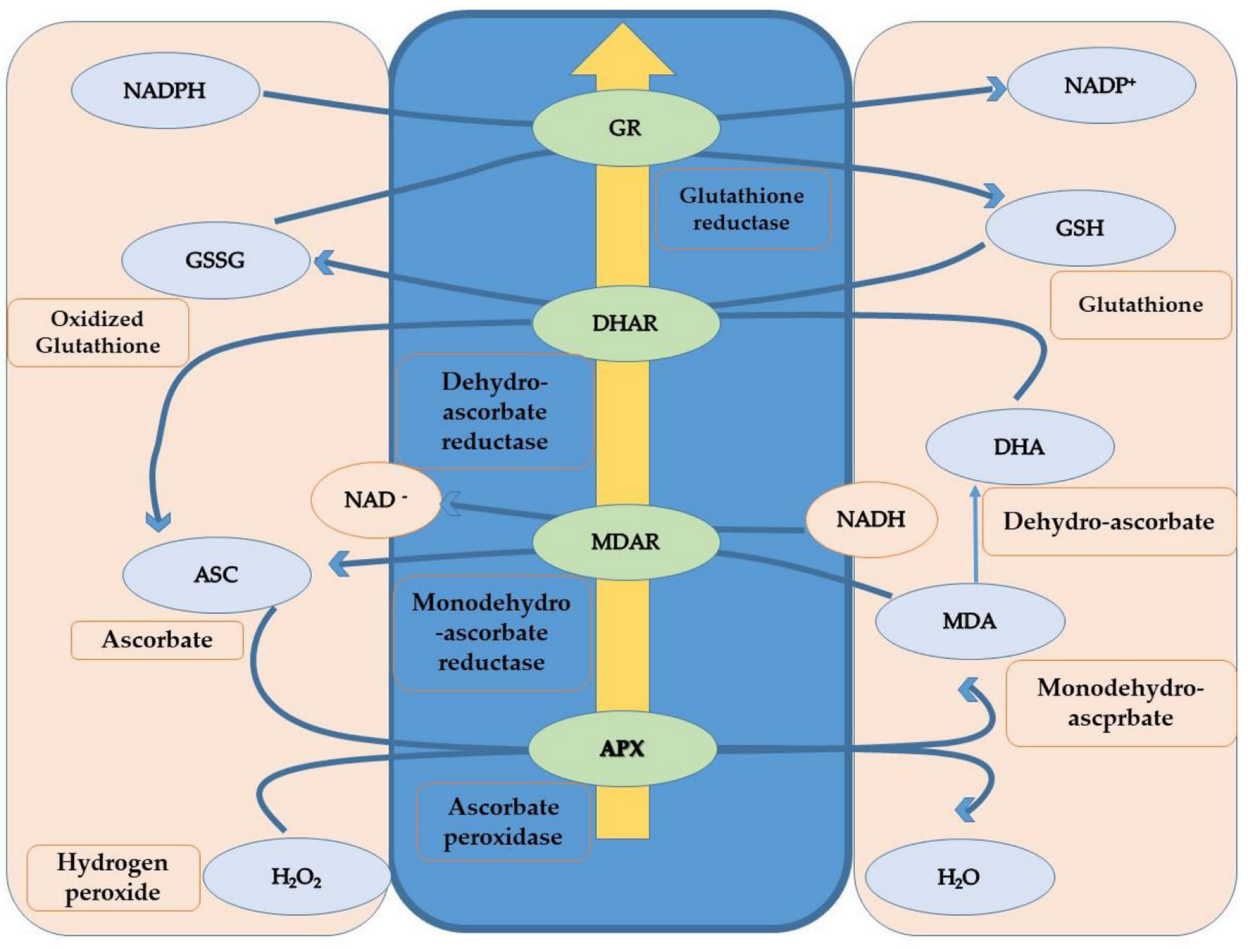
Schematic diagram to show the ASC-GHS cycle to scavenge ROS.

Plants can only survive during unfavorable heat conditions if, somehow, they protect themselves from heat-induced oxidative stress. ROS over-accumulation during stress conditions results in the oxidation damage of vital molecules such as DNA, proteins, and lipids. This condition is termed oxidative stress in plants (Mittler et al., 2012). Plants increase the content of antioxidants through different physiological and biochemical mechanisms to overcome oxidative stress caused by heat stress and scavenge oxygen radicals. Additional enzymes and metabolites participated in the antioxidant defense mechanism. Ascorbate peroxidase (APX), catalase (CAT), superoxide dismutase (SOD), glutathione reductase (GR), glutathione peroxidase, peroxiredoxins, and tocopherols are antioxidant enzymes involved in protecting cells from excess ROS (Lin et al., 2010; Tang et al., 2021). Camelina has shown some resilience to high-temperature stress. However, there is no stability in generating and detoxifying the oxygen radicals under heat stress, as heat stress increases the ROS content. Such an inequity may be the increasing amount of H2O2 under heat stress, which creates oxidative damage in the plant (Ahmad et al., 2021c). The activities of antioxidants (catalase, protease, and ascorbate peroxidase), osmolytes (GB and proline), soluble proteins, and sugars increased under heat stress, which subsequently reduced the H2O2 levels in stressed plants (Sarwar et al., 2018; Ahmad et al., 2021c). Further, thiourea improved the defense system of camellia plants by catalase, protease, ascorbate peroxidase, proline, and glycine betaine activities under heat stress in cotton (Majeed et al., 2019, 2021). Application of thiourea regulated the redox state in plant cells, modulated antioxidant activities, and led to the reduction of lipid peroxidation products (Goyal and Asthir, 2016). Improvement in *B. napus* metabolism due to thiourea application was considered critical to mitigate heat stress damage by regulating photosynthetic pigments and photosynthetic efficiency (Waraich et al., 2021b), which has an important role in redox control during phenological and physiological development and oxidative stress homeostasis (Mhamdi and Van Breusegem, 2018). Non-enzymatic antioxidants marked a decrease in ascorbic acid and total soluble sugars in response to heat stress compared to the non-stressed control (Hameed et al., 2013). In addition, the data also revealed a direct relationship between the activities of antioxidant enzymes (superoxide dismutase, peroxidase, glutathione reductase, ascorbate peroxidase, monodehydroascorbate peroxidase) (Wilson et al., 2014) and the relative expression of genes (heat shock proteins, osmotin, dehydrin, leaf embryogenesis protein, aquaporin), under heat stress (Razik et al., 2021). Seedlings exposed to heat stress with the addition of thiourea significantly improved ascorbic acid content compared to seedlings exposed to heat stress without thiourea (Ahmad et al., 2021a). Irenic improvement in catalase, ascorbate peroxidase, ascorbic acid, proline, and glycinebetaine was observed in response to thiourea supplementation compared to the control (without thiourea application) under heat stress (Catiempo et al., 2021). Proline accumulation is one of the early stress-induced plant responses that acts as a selective trait suitable for assessing abiotic stress tolerance. The ascorbate (AsA) and glutathione (GSH) cycles are fundamental for scavenging ROS (Figure 7). The activation and functions of antioxidants are sensitive to temperature ranges, but their concentration increases as temperature increases. Chakraborty and Pradhan (2011) assert that the concentration of APX (ascorbate peroxidase), SOD (superoxide dismutase), and CAT (catalase) increased at 50°C. Still, the concentration of GR (glutathione reductase) and POX (peroxidase) changes when the temperature ranges from 20 to 50°C in experiments performed using *Lens culinaris*. Consequently, the activity of the antioxidants depends upon the susceptibility and tolerance of plants, time of the season, and their growth stages (Almeselmani et al., 2006). Rani et al. (2013) exposed tolerant and susceptible genotypes of *B. juncea* to the high temperature of (45.0 ± 0.5°C), also observing the high content and activity of POX, APX, CAT, GR, and SOD in tolerant genotypes and less so in susceptible genotypes. Higher concentrations and activities of enzymatic and non-enzymatic antioxidants could be responsible for quenching the reactive oxygen species that help alleviate the negative impact of heat stress-induced oxidative stress.

### CRISPR Technology

Abiotic stresses such as heat, salinity, drought, and waterlogging are critical limiting factors that affect growth, development, seed yield, and quality in oilseed crops (Boem et al., 1996; Purty et al., 2008; Elferjani and Soolanayakanahally, 2018). To date, several mechanisms have been discovered to analyze the mechanism of heat stress tolerance, including overexpression of various miRNAs (Arshad et al., 2017), antioxidant enzymes (Saxena et al., 2020), as well as genes encoding many transcription factors (Hao et al., 2011; Zhu et al., 2018), proteins involved in antioxidant activities (Kim et al., 2019) or osmoprotectants, and proteins facilitating phytohormonal signaling pathways (Sahni et al., 2016) in oilseeds. The success of conventional plant breeding techniques has been extensively studied to regulate heat stress tolerance mechanisms in various crops including oilseeds, but these techniques are very time consuming and cumbersome. As an alternative, genome editing using clustered regularly interspaced short palindromic repeats/CRISPR-associated protein (CRISPR/Cas) has been raised as an innovative technique for precise and efficient genetic manipulations in plant genomes (Subedi et al., 2020). Although, there is a discrete lack of information regarding negative regulators within the heat stress response, and thus studies involving CRISPR/Cas-mediated enhancement of high-temperature stress tolerance mechanisms remain scarce. The multiplex CRISPR/Cas9 system in the regulation of abiotic stress tolerance has been thoroughly reviewed in oilseed rape (Chikkaputtaiah et al., 2017). In this paper, we provide an overview of CRISPR/Cas GE technology in genome editing in oilseed crops, including primary editing (PE), base editing (BE), tissue-specific editing (CRISPR-TSKO), epigenome editing, and inducible genome editing (CRISPR-IGE), which can help to obtain resistant varieties that can tolerate the deleterious effects of high-temperature stress (Chennakesavulu et al., 2021) and has three dimensions, including adoption, crRNA biogenesis, and interference (Gasiunas et al., 2012; Jinek et al., 2012). Synthetic 20-nucleotide guide crRNA or RNA (gRNA) and Cas proteins are introduced into plants via a plasmid. Then, the crRNA or gRNA guides the effector nuclease Cas to identify and alter target DNA sequences in the plant genome. Subsequently, depending on DNA-RNA recognition and cleavage of the designated DNA sequences, CRISPR/Cas technology can be readily designed to induce double-strand breaks (DSBs) at any target site in the genome.

### DNA and RNA Base Editing

Genome-wide studies report that agronomically essential plant traits, including tolerance to abiotic stress, are conferred by introducing these beneficial alleles to one or more single nucleotide polymorphisms (SNPs) in plants, which takes breeders several years (Ren et al., 2010; Li et al., 2015; Singh et al., 2015). The error-free homology-driven repair in plants mediated by CRISPR/Cas allows for accuracy in genome editing by introducing these alleles, but with less effort and efficiency in delivering donor repair templates (DRTs). The discovery of CRISPR/Cas9 technology is a simple, easy, and versatile procedure for genome editing in *B. napus* and *B. oleracea* (Song et al., 2016; Li et al., 2021a). Two groups of base editors have been used (Komor et al., 2016), including the cytidine base editor (CBE), which performs C-G to T-A base substitutions, and the adenine base editor (ABE) is designed for A-T to G-C substitutions. The cytidine base editor consists of a cytidine deaminase (rAPOBEC1) fused to a Cas9 nickase (nCas9) carrying a D10A mutation that inactivates the RuvC domain yet is capable of binding with sgRNA (Komor et al., 2016). Adenine base editors contain an adenine deaminase fused to nCas9 to convert A-T bases to G-C via adenine (A) deamination (Gaudelli et al., 2017). Clustered, regularly interspaced short palindromic repeats (CRISPR) are repetitive and short DNA sequences of 29 nucleotides in length are separated by non-repetitive 32-nt spacer sequences integrated in the anterior portions of protospacer adjacent motifs (Song et al., 2016). Genome editing with CRISPR/Cas9 and its advanced versions have been intensively investigated with many applications: activation or repression of gene expression, gene mutation, and epigenome editing. In plants, the application of CRISPR/Cas9 technology is just emerging due to its high efficiency and simplicity (Song et al., 2016).

### DNA Prime Editing

CRISPR/Cas9 and CRISPR/Cas12a arbitrated genome editing induces a DSB at the targeted sites (Manghwar et al., 2019), leading to unintentional changes or production of abnormal protein(s) due to random insets or removals in the plants. Though genome editing in base editing technology can be performed without double-strand breaks, base editors cannot perform additions, subtractions, and all types of base conversions (Mishra et al., 2020). To overcome these problems, a new approach to genome editing based on CRISPR, called primary editing (also known as genome editing by search and replace), has been discovered (Anzalone et al., 2019), because this approach can write new genetic information by allowing all 12 base-to-base conversions, adding and removing desired nucleotides (up to 44 bp, respectively, 80 bp) in the plant genome without the need for double-strand breaks or donor DNA templates.

### Epigenome Editing

Epigenetic editing includes DNA methylation and histone modifications and controls a plethora of critical procedures in plants, including stability of the genome, imprinting of genes, and expression of different genes under stressful environments (Zhang et al., 2018). Abiotic stress induces histone modifications and hyper/hypo-methylation of DNA, resulting in pressure inducible genes' activation or repression (Sudan et al., 2018). Song et al. (2012) reported that DNA methylation and histone modification might have a mutual effect on the stress-responsive genes in soybean.

### Tissue-Specific Gene Knockout (CRISPR-TSKO)

Essential cellular functions, including growth and development and reproduction, depend upon some highly essential gene families. The removal or absence of these gene families might negatively impact plant performance or even become lethal to the plants (Lloyd et al., 2015). Hence, assessing the role of these genes in plants has rarely been undertaken to date (Lloyd et al., 2015). Researchers discovered a new genome-editing technique known as CRISPR-based tissue-specific knockout (CRISPR-TSKO) (Decaestecker et al., 2019). The Cas9 protein under CRISPR-based tissue-specific KO is expressed in the cell/tissue-specific promoter which leads to the spatial and temporal regulations of gene editing (Decaestecker et al., 2019).

### Tissue Culture-Free Genome Editing

The delivery of CRISPR/Cas9 cassettes is often required in plant genome editing of the explant or recipient tissues in a culture which needs to be treated with many exogenous plant hormones to distinguish them in a whole plant that is expensive, time-consuming (Hiei and Komari, 2008), and only suitable for a limited number of species. The complex process of genome editing has been simplified successfully due to the introduction of new tissue culture methods or, in some cases, avoiding the step of tissue culture. In light of this new technique, the gene-edited somatic cells are reprogrammed into the meristematic cells by the co-expression of developmental regulators (DRs) with genome editing machinery (Maher et al., 2020) that helps to make genome editing faster and more straightforward.

### Inducible Genome Editing (CRISPR-IGE)

The examination of gene functionality largely depends upon plant phenotypic analysis of the loss of function mutants. The knockout or mutations of several genes can be lethal for the plants during different growth stages throughout the life cycle (Lloyd et al., 2015). Therefore, the development of non-viable phenotypes hinders the comprehensive analysis of such vital genes. Whereas cell or tissue type-specified genome editing methods, like CRISPR-TSKO, exist for plants (Decaestecker et al., 2019), there is no method to eliminate a gene in a specific cell or tissue type in a conditional way. Recently, a new and inducible genome editing (IGE) technique has been discovered by merging CRISPR/Cas9 and a well-known XVE (LexA-VP16-ER) inducible technology (Wang et al., 2020). For instance, a heat shock-inducible CRISPR/Cas9 (HS-CRISPR/Cas9) system has been discovered by researchers to generate genetic mutation. The soybean heat-shock protein (GmHSP17.5E) gene promoter and rice U3 promoter (Czarnecka et al., 1989) were used to express Cas9 and sgRNA, respectively (Nandy et al., 2019). Thus, genome editing in Cas9 can only be performed after inclusion with exogenous heat-shock treatment.

### DNA-Free Genome Editing

In CRISPR/Cas technology, target specificity has been provided by the Cas protein and sgRNA. These molecules are usually integrated in the plant genome through the biolistic approach or agrobacterium-mediated transformation technique. The unanticipated changes in the genome created by particular transgenes causes difficulty in upholding a stable phenotype of edited plants (Woo et al., 2015). Furthermore, the presence of CRISPR components in crop plants for a prolonged period may enhance off-target effects; therefore, the transgenes must be eliminated from the plant genome (Woo et al., 2015). Researchers have developed a new approach to solve this problem where rather than recombinant plasmids, preassembled gRNA-Cas9 protein ribonucleoproteins (RNPs) are delivered into the protoplast or *in vitro* zygote (Woo et al., 2015) via a gene gun or transfection. Consequently, the gRNA can direct Cas9 to simplify targeted gene editing without integrating a transgene (Woo et al., 2015), and endogenous proteases degrade Cas9 proteins in plant cells to minimize off-target effects.

The ERA1 (enhanced response To ABA1) and FTA (farnesyl transferase A) genes encode the α and β subunits of farnesyltransferase, which plays a role in ABA signaling, and transformation of these genes leads to hypersensitivity to abscisic acid and reduced stomatal conductance and transpiration rate (Allen et al., 2002; Wang et al., 2009). Stress-induced mutation of both BnERA1 and BnFTA genes in canola has been found to improve the proximity of yield protection under stress conditions (Wang et al., 2005). In addition, in allotetraploid cotton, simultaneous mutation of two paralogous GhARG genes mediated by CRISPR/Cas9-based non-homologous end-joining led to plants with high nitric oxide content and better lateral roots (Wang et al., 2017). For example, the salt overly sensitive (SOS) pathway consists of three major components: the protein kinase SOS2, the calcium-binding protein SOS3, and the plasma membrane Na+/H+ antiporter SOS1 (Zhu, 2002; Guo et al., 2004). Under conditions without a limiting growth environment, gigantea (GI), which is mainly associated with photoperiodic control of flowering and is a major component of stress tolerance (Ke et al., 2017), fixes SOS2 and arrests SOS1 activation. Other potential candidates for NHEJ-based CRISPR/Cas regulation of stress tolerance are specific members of the stress associated protein (SAP) gene family of oilseed species, which have A20/AN1 zinc finger domains and are often differentially defined under stress conditions (Xuan et al., 2011; Dixit et al., 2018). Overexpression of some SAP genes induces widespread improvement in stress tolerance in many plant species (Dixit et al., 2018; Zhang et al., 2019).

### Omics–A Fundamental Approach in Plant Breeding to Improve Abiotic Stress Tolerance

**Figure 8:**
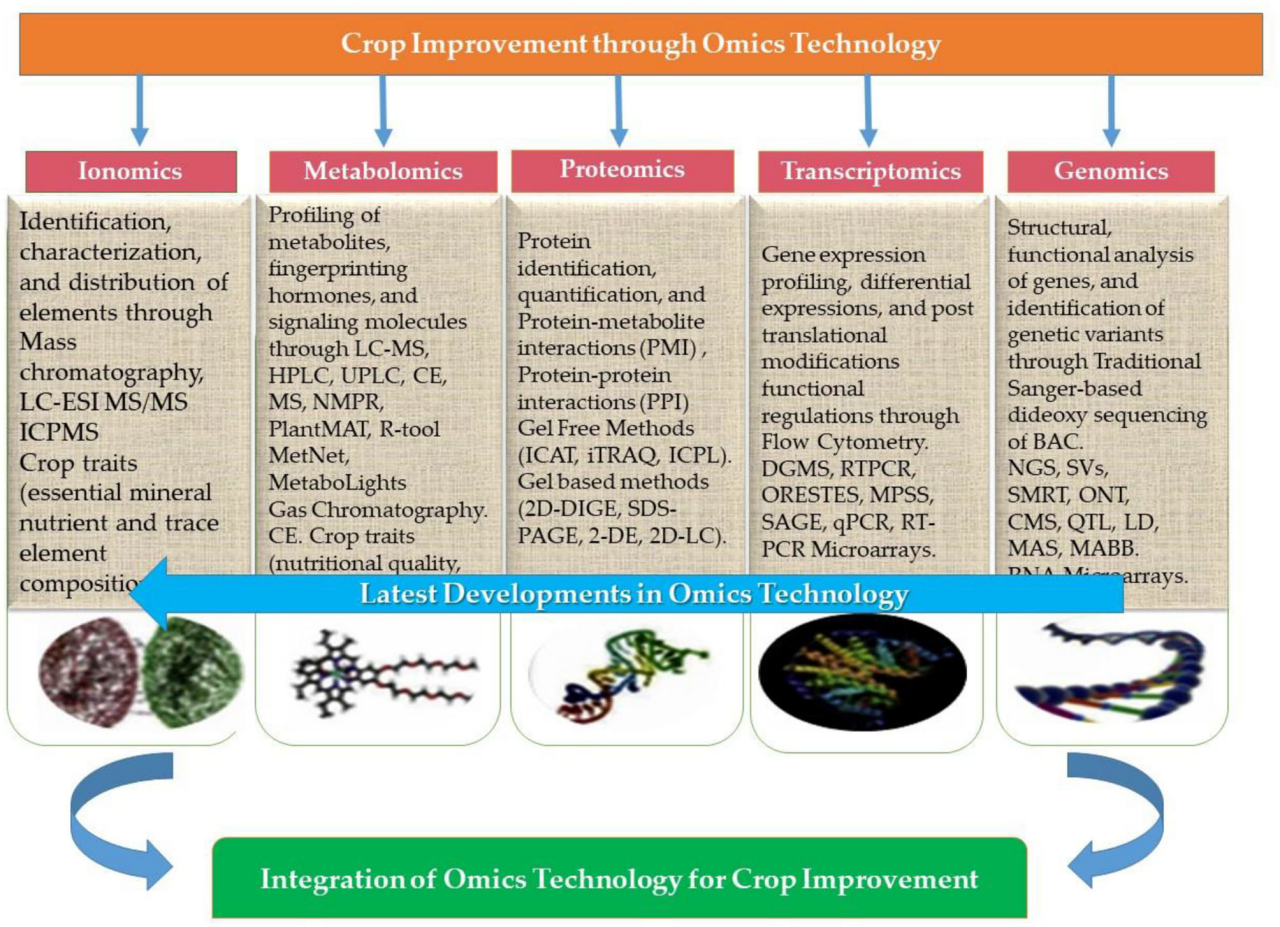
Integration of omics approaches (genomics, transcriptomics, proteomics, metabolomics, and ionomics) for crop improvement (modified form of Zargar et al., 2016).

For several decades, scientists have focused on improving the outcome of significant crops under an ever-increasing abiotic stress environment. Even though the demand for oilseed crops has increased at a rapid rate, researchers never gave much attention to oilseed crops that can ensure food security and nutrition. This part of the article is focused on a fundamental breeding approach aiming to improve the performance of oilseed crops under abiotic stress. Therefore, the importance of omics technology in this context is peerless. Presently, almost 80–85% of rapeseed and soybean reference genomes have been sequenced (850 and 950 megabases, respectively) (Gupta et al., 2017). Similar to these efforts, widespread omics datasets have become available from different seed filling stages in other oilseed crops. Transcriptomic and proteomic studies have detected the majority of starch metabolism and glycolysis enzymes as the possible cause of higher oil in *B. napus* compared to other crops (Gupta et al., 2017). However, gaining insights through discrete omics approaches will never be sufficient to address research questions, whereas assimilating these technologies could effectively decode gene function, biological pathways and genome structures, and the metabolic and regulatory networks underlying complex traits. Hence the integration of omics technologies namely genomics, transcriptomics, proteomics, phenomics, ionomics, and phenomics, has a vital role in crop improvement (Figure 8).

### Conventional Breeding Strategies

The main objective of traditional breeding is to develop high-yielding cultivars under normal environmental conditions. So, the determination of breeders leads the world to produce high-yielding varieties to enhance overall agriculture production (Warren, 1998). High-temperature stress due to climate change may affect the productivity of oilseed crops. Different investigations have been made by breeders and physiologists to develop heat stress tolerance traits in oil crops. They found that these traits are complicated to create because several genes are involved in controlling one specific feature (Blum, 1988). Therefore, stress quantification has a lot of serious issues. In field studies, natural selection is a tough job because of a number of environmental factors that could ruin the accuracy of what is needed for the breeding program. Under field conditions, heat stress is not a consistent phenomenon; it might occur or not which could mislead the breeders to find or develop a resistant trait (Driedonks et al., 2016).

Under a stressed environment scenario, genetic engineering is one of the best economic approaches to develop heat stress tolerance (Blum, 1988). The assessment, identification, characterization, and manipulation through genetic engineering for heat tolerance traits must be evaluated individually for the specific stages through the ontogenesis of crop plants. The heat sensitivity also varies among different species. One example of this is the changes in temperature threshold for groundnut at different growth stages ranging from germination (14–41°C), vegetative development (29–33°C), and reproductive growth (22–28°C) (Prasad et al., 1999, 2000) showing that reproductive growth is more sensitive to heat stress. Plant breeding has advanced to develop tolerant lines for heat stress in many crops, but the range of tolerance and genetic basis still needs to be revealed. The process of the development of new varieties is very costly and time-consuming; therefore, understanding the tolerance mechanisms might help to develop strategies for germplasm screening for the traits which are related to heat tolerance in different oilseeds. Some efforts have been made to build heat tolerance in oilseeds in recent times, for instance, in sunflower (Senthil-Kumar et al., 2003) and cotton (Rodriguez-Garay and Barrow, 1988). Breeders will be encouraged, if the availability of potential donors is good, to deploy such innovative sources in breeding directly but also to exhume the most robust alleles that have the ability to tolerate stress. Consequently, breeding mechanisms for thermo-tolerance are a new approach, and will require a lot of attention in the future. Nonetheless, if the objective is to speed up the progress in the breeding section, most of the emphasis must be placed on (i) the development of a precise/proper procedure for screening; (ii) identifying and characterizing thermo-tolerant genetic resources; (iii) every stage of growth and development of the plant on a genetic basis must be discerned; and (iv) for the transfer of tolerant genes to commercial crops, one must screen and prepare a vast breeding population (Siddique et al., 1999). Progressive molecular biology techniques might enable the development of plants with better thermo-tolerance.

### Quantitative Train Locus

The breeders identified several tolerant genes and their inherent patterns through traditional breeding programs (Wahid et al., 2007). Conventional breeding and transgenic approaches helped us to understand the multi-genic trait phenomenon of heat stress tolerance. Multiple genes control different components, which are very important for heat tolerance in some other tissues and growth stages of the plant (Howarth, 2005). Current developments in genotyping assays and marker discovery set the basis toward the accurate chromosomal positioning determination of QTLs accountable for the heat tolerance in plants (Maestri et al., 2002). The discovery of QTL-linked markers empowers the breeding of stress tolerance pyramiding/uniting of QTL-associated tolerance to different stresses. Numerous major or minor QTLs and associated markers for thermo-tolerance have been recognized in oilseed crops such as groundnut (Selvaraj et al., 2009), sesame (Wang et al., 2016b), and soybean (Guo et al., 2010). Jha et al. (2014) prepared and summarized a list of QTLs linked with heat resistance of many crops with details of the total number of QTLs discovered, mapping of used population, PVE, positions of chromosomes, and associated markers. There are a number of proteins identified which are influenced by heat stress and cause floral abortion, including the cytochrome P450 family, associated with a reduction in the number of silique and abortion of pollens. Adenosine kinase-2 (Radchuk et al., 2006), a protein of pentatricopeptide repeat-containing family (PPR) is linked to the obstruction of flower and pod growth via embryonic abortion, and proteins of the MATE efflux family are associated with embryo development (Zhao et al., 2015). Embryonic and seed abortion-related proteins were also found like pyruvate kinase family protein (Radchuk et al., 2006), phosphatidic acid phosphohydrolase 2, lysine methyltransferase family protein, RGA-like protein 3 (Fischinger and Schulze, 2010), and phosphoenolpyruvate carboxylase 3. There are a few candidate genes that have been identified which were associated with QTLs under artificial heat stress conditions for different oilseed traits.

### QTL Mapping/Linkage Mapping and Linkage Disequilibrium (LD)/Genome-Wide Association Mapping

For QTL mapping, one must have the genomic resources in the shape of genome maps and molecular markers and genetic resources in the condition of the bi-parental mapping population. For the major oilseed crops, some genome maps and molecular markers have been identified (Sun et al., 2007; Xia et al., 2007; Chen et al., 2014; Wang et al., 2016b; Talukder et al., 2019). QTL mapping has been adopted for the genes of complex traits in a number of oilseed crops, for seed and oil yield (Shi et al., 2009) and abiotic stress tolerance (Kiani et al., 2007). An alternate approach of QTL mapping which is now being used in crop science known as LD-based association mapping (AM) was utilized in genetic studies of humans in the early days of its inception. The basis of AM in the germplasm collection is the correlation between phenotype and genotype. The use of AM in QTL detection has many advantages on bi-parental linkage mapping, such as (1) manipulation in all events of recombination that happened in the crops' evolutionary history that resulted in the much-advanced resolution of mapping; (2) in this case, there is a need to prepare a particular population that shortens the time required in QTL mapping, and (3) as linkage mapping is suitable for the study of only two alleles, AM can detect/study a higher population of alleles (Neale and Savolainen, 2004). However, AM has its drawbacks, including a specious/false-positive linkage between a trait and a marker. Many statistical tools have been developed to address the problem of hidden population structures (Falush et al., 2003). The leading causes for these problems are mating systems, genetic drift, and incorrect selection (Flint-Garcia et al., 2003). The genome-wide association mapping system under high-temperature stress is being used in several oilseed crops like soybean (Li et al., 2014), rapeseed (Cai et al., 2014; Zhu et al., 2017), cotton (Sun et al., 2019), sunflower (Dowell et al., 2019), groundnut (Jiang et al., 2014), and sesame (Wei et al., 2013).

### Transcriptomics: A Key to Understanding Abiotic Stress Responses in Plants

The study of the transcriptome from a specific tissue, a particular organ, or organism under specified circumstances is known as transcriptomics. Transcriptomics characterizes the transcriptome as a whole where all the expressed genes have been studied at one term, underneath a particular physiological condition or developmental stage. It paved the way to understand how the plant responds to abiotic stresses. The transcriptomic approach is much more complicated than the genome that encodes it as a number of the same types of mRNA can be produced by one gene that encodes various proteins through alternate splicing. In *B. napus*, the levels of DNA methylation increased more in a heat-sensitive than a heat-tolerant genotype under heat stress (Gao et al., 2014). Transcriptomic analysis by next-generation sequencing (NGS) and RNA–seq for sRNAs has primarily improved genomic resources since it was used in genomics research (Ulfat et al., 2020). In contrast to the past, sequencing-based and hybridization-based approaches can help understanding of the gene expression of multiple genes at a time at the whole-genome level. Microarray technology is a leading technology in hybridization-based methods that was used in oilseed crops for large-scale gene expression, for example, *B. napus* (Raman et al., 2012), sunflower (Fernandez et al., 2012), soybean (Ding et al., 2021), and groundnut (Guo et al., 2011; Xiao-Ping et al., 2011). We have better alternatives of gene expressions through sequence-based approaches like serial analysis of gene expression (SAGE), expressed sequence tags (ESTs), open reading frame EST (ORESTES), and digital expression analysis (RNA-seq) by utilizing generation sequencers and massively parallel signature sequencing (MPSS) (Marioni et al., 2008; Campobenedetto et al., 2020). Whole-genome RNA-seq became more convenient, and the gene expression at the whole genome level was rapid due to the emergence of NGS. This is helpful in organisms with a limited genome and some non-model lacking reference genes (Strickler et al., 2012). RNA-seq has been used in many oilseeds like in canola (Jiang et al., 2013), soybean (Kim et al., 2011; Ding et al., 2020), oil palm (Shearman et al., 2013), sunflower (Fass et al., 2020), and groundnut (Chen et al., 2013). Phylogenetic, collinearity, and multi-plesynteny analyses exhibited dispersed, segmental, proximal, and tandem gene duplication events in the HSF gene family. Duplication of gene events suggests that the HSF gene family of cotton evolution was under strong purifying selection. Expression analysis revealed that GhiHSF14 is upregulated in heat stress in cotton (Rehman et al., 2021). The microarray data characterize numerous tissues, developing stages, and ecological situations as shown in Table 2.

**Table 2:** Omics studies on heat stress tolerance in different oilseed crops.

**Crop**	**Temperature (°C)**	**Omics techniques**	**Plant part**	**Method**	**Trait/treatment stage**	**No. of proteins/genes differentially identified**	**Location**	**References**
Soybean	42	Genomics	Seeds	RT-PCR, qRT-PCR analyses	HSF family genes	38	China	Li et al., 2014
Canola	40	Genomics	Seeds	RNA-seq and qRT-PCR analysis	HSF gene family	64	China	Zhu et al., 2017
Soybean	35	Transcriptomic	Seeds	RNA-Seq analysis	Biostimulant mechanism	879	Italy	Campobenedetto et al., 2020
	38/32	Transcriptomic	Male organ	Real-time PCR (qRT-PCR)	Cytoplasmic male sterility (CMS)-based hybrid (F1)	8,784	China	Ding et al., 2020
	38/32	Transcriptomic	Male organ	qRT-PCR	Cytoplasmic male sterility (CMS)-based hybrid (F1)	1,145	China	Ding et al., 2021
Sunflower	45	Transcriptomic	Seedlings	qRT-PCR	Phenological traits	97	Argentina	Giacomelli et al., 2012
Cotton		Transcriptomic	Seeds	Multiple sequence alignments (MSA), NA-seq expression	Cis-regulatory elements	79	China	Rehman et al., 2021
Canola	40/30	Proteomics	Leaf	RPLC, LC-MS/MS	Carbohydrate metabolism, HSPs, and chaperones	1,022	China	Yuan et al., 2019
Soybean	37	Proteomics	Anther	SDS-PAGE	Reproductive organs	371, 479, and 417	China	Li et al., 2020
	40	Proteomics	Roots	LC-MS/MS	Root hairs and stripped roots	1,849 and 3,091	USA	Valdés-López et al., 2016
Sunflower	33/29	Proteomics	Leaf	HPLC	Reproductive stage	2,343	Spain	De La Haba et al., 2020
Soybean	42/26	Metabolomics	Seed	UPLC/MS/MS2, UP LC/MS/MS2, GC/MS	Oil	275	USA	Chebrolu et al., 2016
	43/35	Metabolomics	Leaf	UPLC/MS, GC/MS				Das et al., 2017
Canola	31/14	Metabolomics	Floral buds	Gas chromatography–mass spectrometry GC–MS	Heat	25	Canada	Koscielny et al., 2018
Soybean	45/28	Phenomics	Leaf	OJIP protocol of a Fluorpen Z995-PAR	Vegetative (4th leaf stage)		USA	Herritt and Fritschi, 2020
	42/28	Phenomics	Leaf	PAM fluorometer, SPAD	Germination		India	Jumrani et al., 2017
Brassica	35/25	Phenomics	Leaf	Scanalyzer, LC, PRI, Qy	Reproductive stage		Australia	Chen et al., 2019
Cotton	38	Phenomics	Leaf	IRGA, Spectro-photometer	CMT, CSI		USA	Singh et al., 2013

### Proteomics Approach

An emerging technology that can provide a precise and tremendous amount of information regarding various metabolites and proteins generated due to abiotic stresses is proteomics (Rodziewicz et al., 2014). The role of proteomics is to decipher the importance of redox homeostasis, chaperons or heat shock proteins, proteins essential in signal transduction, and metabolic pathways during heat stress. It endorses the amount of protein present and sends direct information, giving more precise knowledge and a level of understanding compared to genomics. The sustainability and crop improvement in oilseeds can be achieved by integrating proteomics and genetic data of root systems under high temperatures in oilseeds (Valdés-López et al., 2016). The primary problem of proteomics is the presence of multiple genes at one time that have gone through PTMs.

Nevertheless, this technology is emerging fast with a principal focus on protein interactions, protein quantity, and post-translational modifications (Champagne and Boutry, 2013). Generally, proteomics can be used for proportional expression analysis of two or more protein samples, for understanding post-translational variations, for proteome profiling to recognize how proteins perform biological progressions, for learning of protein-protein relations, and for ascertaining novel biomarkers to sense and screen exact stress expressions (Chandramouli and Qian, 2009). Several significant experiments have been conducted using the proteomics approach in oilseed crops (Table 2). In oilseed crops, the best way to improve stress tolerance is to associate different candid proteins that are physiologically significant. In oilseed crops, proteomic identification has been made by using both the gel-free and gel-based proteomics approaches (Chandramouli and Qian, 2009), and separation is done by using the most frequently used gel-based strategies, including one-dimensional gel electrophoresis (SDS-PAGE) (Han et al., 2013; Messaitfa et al., 2014; Li et al., 2020) and 2D-polyacrylamide gel electrophoresis (2-DGE) (Ghaffar et al., 2020). MS techniques like ESI and MALDI TOF proteomic-based experiments are very accurate and more precise due to the accessibility of many genomic sequences of many organisms and EST information. Though, it has some technical issues like restricted dynamic resolutions when a substantial number of proteins are drawn to analyze the identification and separation of hydroponic proteins and obtainability of the pure proteome. These challenges can be overcome by using fluorescent dyes, application of affinity chromatography, reverse phase HPLC, and explicit fractionation techniques. Besides, we can characterize the complete proteome through high-throughput techniques (i.e., robotics, spectrometers, and multi-dimensional chromatography). These valuable tools have already been tested. For instance, the functions of different proteins have been evaluated using proteomics in other plants like soybean (Mooney et al., 2004; Natarajan et al., 2006). In addition to all that, new gel-free, highly efficient methods have been developed for proteomic analysis. This discovery opens the possibility of identifying many genes and replacing the low-throughput techniques. The gel-free techniques currently used for oilseed crops include ICAT (isotope-coded affinity tagging) (Oh et al., 2014), MudPit (multidimensional protein identification technology) (Agrawal and Rakwal, 2008), iTRAQ (isobaric tagging for relative and absolute quantitation) (Li et al., 2020), and SILAC (stable isotope labeling by amino acids in cell culture) (Zargar et al., 2013). The novel techniques of the long-column method, 2D-LC, and iTRAQ OFFGEL fractionation, have been developed to identify low abundance proteins. Progressive automatic peptide purification systems with great accuracy and more reproducibility are a crucial task in plant proteomics (Zargar et al., 2013). Additionally, iTRAQ is more precise and consistent for protein quantitation than traditional 2-DE analysis (Qin et al., 2013).

### Metabolomics Approach

The genes and proteins that play a crucial role in plant stress responses are identified using genomics, transcriptomics, and proteomics. The boundary of metabolic pathways and regularity networks responding to a specific stressor or a number of simultaneous stresses is needed for the proper understanding of stress response in plants. A new zenith has been provided by metabolomics for stress-related studies in crop plants and has become a crucial tool to understand the molecular mechanisms underlying stress responses (Weckwerth and Kahl, 2013). Targeted organism's metabolomics is a non-biased, comprehensive, and high-throughput analysis of the complex metabolite mixture. This is an important technique that, in collaboration with genomics, transcriptomics, and proteomics, can provide a missing link in functional genomics, offer new insights into the study of systems biology, and can more accurately elucidate biological mechanisms (Saha et al., 2019). With further advances in proteomics, metabolomics is a dynamic technique to functional genomics that allows us to recognize and quantify metabolomes within a single cell, organ, or organism (Chebrolu et al., 2016). Plant metabolism undergoes specific configurational changes to achieve metabolic homeostasis. It synthesizes different compounds to mitigate the adverse effects of any stress it may experience in its life cycle. There have been significant advances in metabolomics that could provide greater insight into the various mechanisms of thermo-stress tolerance at the metabolic level (Bokszczanin and Fragkostefanakis, 2013). For example, in studies on soybean, multiple antioxidants have been found to play a role in improving thermo-tolerance through metabolite studies (Chebrolu et al., 2016). Metabolomics techniques include some separations approaches like HPLC, capillary electrophoresis (CE), gas chromatography (GC), mass spectroscopy (MS), ultra-performance liquid chromatography (UPLC) along with detection techniques like nuclear magnetic resonance (NMR) (Das et al., 2017). In metabolite studies, one has to be focused on all the metabolites at one time because metabolism in plants is very dynamic and every single aspect might be linked with some other metabolites with different expressions; moreover, it might produce multiple metabolites at certain times (Fischbach and Clardy, 2007). Several key proteins involved in seed storage proteins, fatty acid metabolism, allergens, and toxins connected with the development of castor oil seeds were recognized by engaging an isobaric tag for relative and absolute quantification (iTRAQ) and isotope-coded protein labels (ICPLs) and technologies. Understanding the major metabolites in soybean plants in response to high-temperature conditions can help in the development of heat-resistant varieties. The concentrations of flavonoids, ascorbate (AsA) precursors, and tocopherols were higher in heat-tolerant genotypes than in heat-sensitive ones (Feng et al., 2020), and these metabolites can alleviate the adverse effects caused by damage from heat-induced reactive oxygen species (ROS) during seed maturation under high temperatures (Das et al., 2017). Many studies have shown that ROS-scavenging mechanisms play an important role in protecting plants from heat stress (Xu et al., 2016).

### Ionomic Approach

This refers to an omics study that deals with all the quantitative documentation of the whole set of ions in an organism under various external stimuli and then quantifies the changes in ion production. The production of these ions can be elucidated by different biochemical pathways that play a vital role in mineral transport—enzyme catalysis is a cofactor in some important regularity pathways—and maintaining the integrity of the cell. Therefore, any variation or change in the process of ion production results in serious changes in metabolic processes. The plant ionome must be studied to understand the key role of ions to carry life processes. Therefore, a good understanding of gene regulation can be achieved by ionomic-based studies. These ionomic studies are known to differentiate natural alleles and different mutants (that might have variation in one or several elements) (Chen et al., 2009). Ionomics is very important to understand the elemental composition profile and their role in the nutritional requirement and physiological and biochemical functionality. It was observed that these two genes alter the ionome and elements that present in the leaf. So, these variations caused the shift in gene expression and change the multi-elemental profile due to variation in water and ion transport (Ziegler et al., 2013). Additionally, multi-elemental profiling helps to detect mutants with various numbers of ions in soybean seeds (Ziegler et al., 2013). The plant elemental profile is controlled by a number of factors like availability of elements, their uptake, their transport, and external conditions that carry out evapotranspiration. These factors have made the ionome plan very specific and very sensitive so that the elemental composition shows several different states. Silicon is found to have a good role in the abiotic stress tolerance in plants (Liang et al., 2007) but soybean cannot accumulate silicon due to some genetic differences, except recently a silicon transporter gene was introduced in soybean by using ionomic technologies (Deshmukh et al., 2013).

### Phenomics Approach

In recent years, great progress was made in the field of genomics with the objective of understanding and unraveling crop genomes. It incorporates the development of various kinds of genotyping platforms: molecular markers and the study of marker-trait associations lead to the innovation of genes/QTLs, and genetic mapping procedures, which make main crop genome sequences accessible and garners improvements in sequencing technologies, lead to a decline in the costs of sequencing. The sequencing methods have certain improvements that have made the crop genomes and plant sequencing genomes monotonous (Jackson et al., 2011). Additionally, it is possible to identify allelic variation through crop genome sequencing (Furbank and Tester, 2011). Some additional costs of genotyping of the plant genome could be reduced with the advancement in high-throughput markers and genotypic platforms (Jumrani et al., 2017).

**Figure 9:**
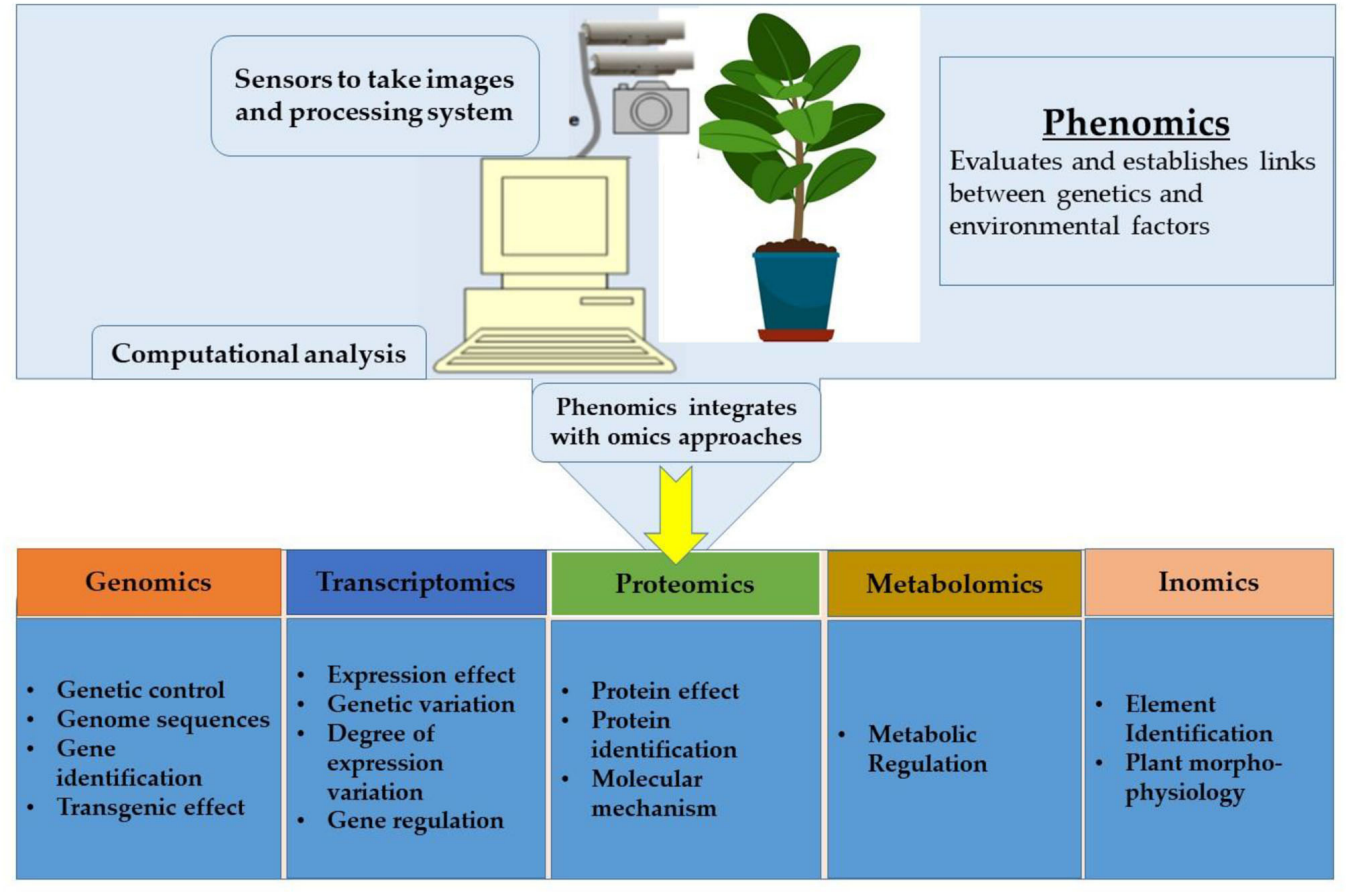
Phenomics and its integration with other omics approaches (adopted from Deshmukh et al., 2014).

Furthermore, for the more accurate results from the other omics techniques like proteomics, transcriptomics, genomics, etc., one must link their information with proper phenotyping. The bottlenecks in phenotyping are (i) phenotyping in replicated trials in numerous situations over years; (ii) sluggish and expensive phenotyping; (iii) for QTL/gene discovery, the phenotyping of large mapping populations, followed by the cloning of significant QTLs; (iv) less accurate approximations of phenotypic data for testing allelic disparities of a candidate gene in a germplasm set; and (v) destructive tools used in phenotyping at static times/growth stages (Furbank and Tester, 2011). All of these are responsible for the gap created between genotypes and phenotypes referred as the GP gap. For the resolution of this problematical bottleneck, the phenomics revolution is the need of the hour. Efforts are made worldwide to overcome this problem by evolving plant phenomics amenities that can scan and measure data for hundreds of thousands of plants in a day in a cultured way (http://www.plantphenomics.org.au/). These efficient phenomics amenities make use of good non-invasive imaging, image analysis, spectroscopy, high-performance computing facilities, and robotics hence saving labor, cost, and time. Consequently, combined with all other omics approaches, phenomics has a considerable future in plant breeding and genetics (Figure 9).

## Agronomic Approaches

### Nutrient Applications

High-temperature stress that could lead to nutritional deprivation is a significant factor contributing to impaired plant growth and development. At the same time, exogenous application of nutrients may alleviate the negative impacts of heat stress coupled with fulfilling the nutritional requirement. Studies have revealed the ameliorating effects of nutrient applications. Nevertheless, foliar or extracellular application of nitrogen (N) and potassium (K) may improve the ability of the plant to tolerate high-temperature stress (Hammac et al., 2017; Muhammad et al., 2019). The application of micronutrients, such as Se (selenium), B (boron), Mn (manganese), and macronutrients, such as nitrogen (N), potassium (K), sulfur (S), and calcium (Ca^2+^) can modulate leaf water status, stomatal regulation, and upregulation of physiological and metabolic processes that increase heat stress tolerance (Waraich et al., 2012). Seed priming with potassium nitrate played an important role in mitigating heat stress by increasing the concentration of nitrate reductase, catalase, peroxidase, proline enzymes, and chlorophyll content, which helped sesame to maintain its performance under stress conditions (Kumar et al., 2014, 2016). High-temperature stress reduces net carbon gain and dry matter production in soybean under both P application and P deficiency conditions and reduces net carbon gain (Singh et al., 2018). Increased S supply has been shown to lead to higher levels of total glucosinolates in *Brassica rapa* (Li et al., 2007) and individual glucosinolates such as glucoraphanin and glucoraphasatin (Krumbein et al., 2001), sinigrin, glucobrassicanapin, gluconapin, and progoitrin in *Brassica juncea* (Kaur et al., 1990), which helped induce heat tolerance at elevated temperature. In addition, the application of sulfur at high temperatures increased the activity of various enzymes, including nitrate reductase, glutamine synthase, and glutathione dehydrogenase, which are essential in nitrogen metabolism in sunflower (Ahmad et al., 2020). Foliar-applied sulfur alleviated the deleterious impact of high-temperature stress in canola by increasing plant gas exchange attributes including photosynthesis and stomatal conductance which depends upon the water status in the plant cells and regulates the gaseous exchange to improve yield and yield components in camelina (Waraich et al., 2021b). In oilseed crops, the role of sulfur is undeniable as it helps to improve the seed quality parameters. In contrast, in the absence of sulfur, the seed oil content decreased in oilseed crops under average or heat stress conditions (Brunel-Muguet et al., 2015). However, sulfur with expected thermo-sensitization effects may also have the impact a few nutritional (fatty acids, seed storage protein concentration) and physiological (IAA, SA, ABA: GA3 ratio) quality criteria, as well as the antioxidant capacity in *B. napus* (de Almeida et al., 2021).

### Plant Growth Regulation

**Figure 10:**
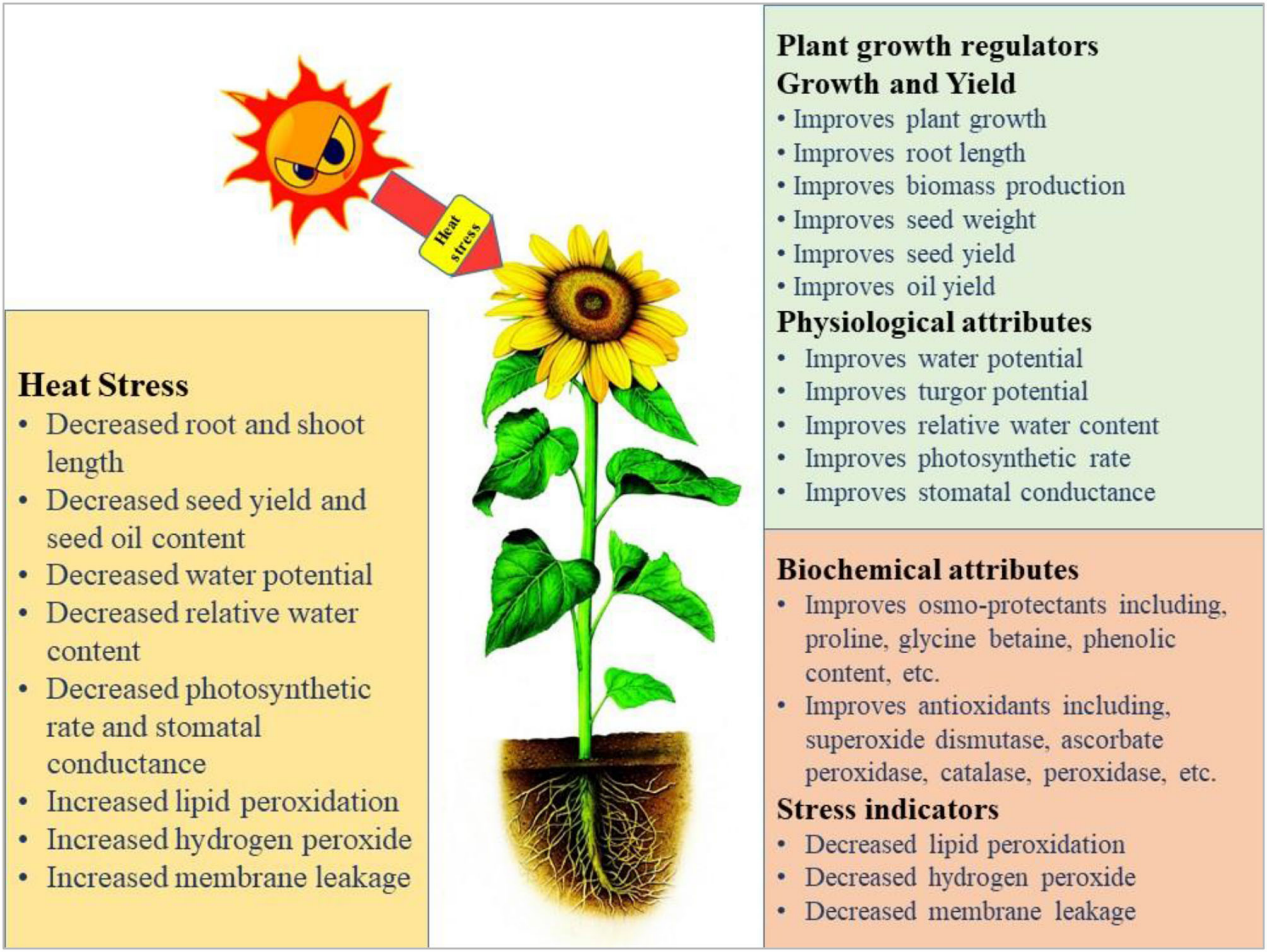
Effect of heat stress and application of plant growth regulators on phenological, physiological, and biochemical properties of oilseed plants.

With a naturally induced defense system, many chemical compounds regulate the plant activity under heat stress at physicochemical levels (Ahmad et al., 2021a). To improve the growth and productivity of oilseed crops under environmental stress, the supplementation of plant growth regulators (PGRs) either through seed or on foliage holds a superior position. The plant growth regulators are chemicals that may regulate the growth, physicochemical attributes, and stress tolerance mechanisms under stressful environments (Shah et al., 2021). Thiourea, as a plant growth controller, may mediate plant growth under high-temperature stress. Thiourea (TU) is a growth promoter under stress conditions due to its redox regulatory property imparted by the –SH group and regulates the cell homeostasis to induce stress tolerance (Sahu, 2017; Wahid et al., 2017). Waraich et al. (2021a) revealed the role of thiourea to upregulate the gas exchange and water relations in camelina genotypes grown under high-temperature stress. The results of the current study showed that application of thiourea (applied either at the vegetative or reproductive stage) improved the growth and yield under heat stress by maintaining the gas exchange traits, antioxidant enzyme activities, and osmoprotection in sunflower (Akladious, 2014), canola (Ahmad et al., 2021a,c), and camelina (Ahmad et al., 2021b) as shown in Figure 10. Exogenously applied abscisic acid enhances plant defenses by regulating the accumulation of soluble sugars that improve the lipid profile in castor bean (Chandrasekaran et al., 2014), while in *Brassica napus* it increases the accumulation of a synthetic brassinosteroid (24-epi-BL) that induces heat tolerance (Kurepin et al., 2008). γ-aminobutyric acid (GABA) significantly improved the accumulation of osmolytes including proline, soluble proteins, and sugars, activities of antioxidant enzymes (superoxide dismutase, ascorbate peroxidase, glutathione reductase, peroxidase, monodehydroascorbate peroxidase), and relative gene expression (dehydrin, heat shock proteins, osmotin, leaf embryogenesis protein, aquaporin) (Razik et al., 2021), which helped to reduce hydrogen peroxide and malondialdehyde content in GABA-treated plants compared to untreated plants under heat stress with an increase in the levels of gene transcripts encoding antioxidant enzymes, suggesting that GABA regulated antioxidant defenses and could be partly responsible for the improved heat tolerance in sunflower (Razik et al., 2021). Heat stress is one of the causes of gamma-aminobutyric acid (GABA) accumulation in sesame (*Sesamum indicum* L) plants (Bor et al., 2009). Similarly, Bor et al. (2009) reported that a short heat shock interval also increased the endogenous GABA content in pea and sesamum indicum plants. Pre-treatment of soybean seeds with 1 mM of putrescine (Put), spermidine (Spd), and spermine (Spm) alleviated heat stress-induced damage by improving growth parameters and antioxidant defense compared to water-sprayed control (Amooaghaie and Moghym, 2011). Brassinosteroids positively affect plant responses to abiotic stresses by maintaining Na+ homeostasis, metal sequestration, increasing heat shock protein synthesis, enhancing GRX (glutaredoxin) and NPR1 (non-expressor of pathogenesis-related genes 1) for redox signaling, and increasing the activities of enzymes involved in the ascorbate-glutathione cycle (Ahammed et al., 2020).

### Microbial Inoculation

A number of microorganism-based stress mitigation mechanisms have been proposed for plant species. Microbes belonging to different genera of *Achromobacter, Variovorax, Azospirillum, Bacillus, Azotobacter, Enterobacter, Klebsiella, Aeromonas*, and *Pseudomonas* have demonstrated the ability to enhance plant growth even under adverse environmental conditions (Arkhipova et al., 2007) including high-temperature stress (de Zelicourt et al., 2013; El-Daim et al., 2014). Microbial inoculation enhances the regulation of the plant defense system by improving the production of enzymatic and non-enzymatic antioxidants along with the production of osmolytes under high-temperature stress. Endophytic fungus has been found to stimulate vegetative growth and biomass production due to its role in mediating the photosynthetic system including chlorophyll content compared to non-inoculated plants (Ismail et al., 2020). Several microbes have been found to play an ameliorative role at elevated temperature by improving the antioxidant content of plants, viz., *Bacillus tequilensis* (SSB07) was very promising for mitigating the negative effects of climate change on crop production as it improved root/shoot length, biomass, leaf development, the content of photosynthetic pigments, endogenous jasmonic acid, and salicylic acid in the phyllosphere, and significantly reduced stress-responsive ABA overproduction (Kang et al., 2019). The improvement in plant growth was reflected by greater plant height, leaf area, biomass, and photosynthetic pigment production under heat stress and expected conditions in the inoculated plants. Under stress conditions, *Glomus intraradices* and *G. mosseae* were found to improve seed oil content in *B. napus* (Keshavarz, 2020). Application of *Bacillus cereus* SA1 under high-temperature stress enhanced the defense system of soybean plants by increasing superoxide dismutase activity, ascorbic acid peroxidase and glutathione content, and expression of heat shock proteins (GmLAX3 and GmAKT2), which have been linked to reduced detoxification of reactive oxygen species, increased potassium gradients, and altered auxin and ABA stimuli, and which are critical for plants under heat stress (Khan et al., 2020). *Bacillus tequilensis* (i.e., SSB07) improved the growth of Chinese cabbage seedlings and produced the gibberellins GA_1_, GA_3_, GA_5_, GA_8_, GA_19_, GA_24_, and GA_53_, as well as indole-3-acetic acid and abscisic acid. The application of *B. tequilensis* SSB07 was also found to increase the shoot length and biomass, leaf development, and photosynthetic pigment contents of soybean plants. Under heat stress, SSB07 injection significantly increased the endogenous jasmonic acid and salicylic acid contents of the phyllosphere and significantly downregulated the production of stress-responsive ABA. Thus, *B tequilensis* SSB07 shows promise for countering the harmful effects of climate change on crop growth and development (Kang et al., 2019).

### Smart Agricultural Technology

The latest technologies applied to crops can reveal plant responses to various biotic and abiotic stresses. However, the practical application of these technologies is not widespread among stakeholders due to their high cost. Among irrigation techniques, sprinklers, gravity irrigation, subsurface drip irrigation, and center pivot irrigation can cool plants at elevated temperatures, which would be the preferred method for commercial growers to manage crop losses due to heat stress to reduce water losses from traditional irrigation methods. Soil moisture is critical during heat stress. Shade covers, made of lightweight materials or those commercially available in various materials, are an inexpensive strategy applied on a large scale to manage heat stress. In addition, remote sensing technologies that use thermal imaging, satellite imaging, thermal imaging, and hyperspectral sensing are being used to detect heat stress before symptoms are detectable, thus preventing agricultural losses (Hernández-Clemente et al., 2019). An airborne infrared/visible imaging spectrometer was used to quantify heat stress tolerance based on changes in soil surface temperatures (Shivers et al., 2019). The photochemical reflectance index (PRI) measured by aerial hyperspectral scanners reveals the moisture status of crop plants concerning heat stress to allow gradual feeding without adverse effects on proper growth and development (Suárez et al., 2008). New discoveries in remote sensing and plants genomics enable climate-smart agriculture by developing climate-resilient crops (Jumrani et al., 2017; Hossain et al., 2021). Remote sensing techniques may help obtain accurate calibrated measurements of environmental factors that affect the performance of oil crops over a range of spatial and temporal resolutions and thus help sustain agricultural productivity under heat stress. Chlorophyll fluorescence measured using a spectro-radiometer and chlorophyll fluorometer under high-temperature stress may help induce a heat tolerance mechanism in plants in cotton (Van der Westhuizen et al., 2020). Therefore, chlorophyll fluorescence techniques may help in non-invasive eco-physiological studies to access responses of plants against high-temperature stress (Jumrani et al., 2017). However, the fluorometer in the leaf chamber is a pulse amplitude modulation (PAM) fluorometer to measure leaf fluorescence in light and dark-adapted leaves, which can be used to recognize the basis of photosynthesis and plant responses to environmental changes (Khan et al., 2020). Along with physiological observations, plant morphological attributes, such as leaf curling early in the morning at low air temperature, which indicate the onset of high-temperature stress, can be used to identify the negative effects of heat stress in oilseed plants. Curled leaves impair the transpiration rate by reducing leaf surface area, which reduces light interception, affecting the water and nutrition uptake. Cell sap observations in the early morning can also be used to identify the impact of high-temperature stress. However, the crop stage at the onset of heat stress is imperative for determining the type of treatment to alleviate the impact of high-temperature stress. Under the shadow of smart technologies, genome editing (GE) is one of the most powerful techniques to improve heat stress tolerance by manipulating the genome sequence in plants. Genome editing may help improve crop performance under high temperature, and has shown a remarkable potential to tackle the insecurities of the food industry and develop a climate-smart agriculture system globally (Liu et al., 2013). On the other hand, plant nutrition also has an important role in heat stress in oilseeds because foliar spraying of Zn regulated the physiological properties of plants which helped to increase the number of siliques per plant, number of seeds per silique, thousand seed weight, seed yield, seed oil content, and linoleic acid content. In contrast, erucic acid, stearic acid, and glucosinolate were decreased (Rad et al., 2021). However, the improvement of Zn supplementation may increase seed oil content due to the production of auxin biosynthesis, chlorophyll content, nitrogen uptake, phosphorus uptake, and a reduction in sodium concentration in the plant tissues. Another important consideration for the development of climate-smart oilseed cultivars is that the vast majority of studies in which abiotic stress tolerance has been assessed thus far have been based upon the effect of a single form of stress. While prolonged or acute exposure to any single abiotic stress can be enough to devastate oilseed crop yields in the field, several stresses often co-occur in various combinations and at varying levels, which can compound the resulting negative effects (Elferjani and Soolanayakanahally, 2018). The precise molecular effects of these interactions are not well-understood. Therefore, a better understanding of the mechanisms of response to abiotic stresses under complex growing conditions will be fundamental to maximizing our ability to ensure future oilseed improvement using any breeding platform.

## Conclusion and Future Perspectives

Oilseeds are an important source of food for human consumption, and are used as fuel for biodiesel and as various industrial products. Under a climate change scenario, there is a high probability that the temperature will exceed the threshold for oilseeds. Plant responses to heat stress vary from symptomatic to quantitative. Although, the reproductive stage, the outcome of which symbolizes the economic value of oilseeds, is specifically more susceptible to high heat stress, which directly affects the male and female reproductive parts. Lipid peroxidation leading to excessive ROS production, changes in antioxidants, and reconfiguration of metabolite synthesis also plays a significant role. In response to high-temperature stress, a few adaptive mechanisms are manifested in plants, including a wide range of morphological, physiological, and molecular mechanisms that enable plant survival. Physiological and molecular mechanisms are essential to help breeders develop better genotypes that can perform better under heat stress. At present, the physiological mechanisms of heat stress are reasonably well-understood, but more profound knowledge is needed in several areas, particularly to better understand the physiological basis of the source-to-sink partitioning of assimilates. The introduction of signaling cascades leads to profound changes in uncharacterized gene expression that are central to adaptation to heat stress. Although several signaling molecules are activated/expressed at high temperatures, Ca2+ regulation remains critical. Expression of HSPs, HSFs, and other stress-related chaperones that serve to fold and unfold basic proteins under stress confirm the three-dimensional assembly of membrane proteins for sustained cellular function and persistence under high-temperature stress. The potential applicability and popularity of genome editing enables sustainable development of plant resistance to abiotic stress. Although the use and development of CRISPR/Cas-based technologies in oilseed crops is still in its infancy, it is clear that these high-precision molecular breeding tools have the potential to provide unprecedented levels of productivity improvement in agronomically valuable oilseed crops and could thus contribute significantly to our ability to sustainably meet future demand for oilseed-derived products. Omics has gained momentum in the last few decades and has become a tool for crop rescue in the context of climate change. The combination of multi-omics approaches will play a major role in identifying stress-responsive genes and identifying the role of different genes in metabolic pathways and the use of this information in the rapid development of climate-resilient oilseeds. Thus, the application of genomics, transcriptomics, proteomics, phenomics, and ionomics approaches seems to be more appropriate to better understand the molecular basis of oilseed response to heat stress in addition to plant tolerance to heat stress. Study evolution is expanding the gene pool by using advanced biotechnological tools using omics, which is the best way to increase productivity. The CRISPR/Cas9 genome editing system and omics technologies promise a future for agricultural biotechnology in sustainable improvement of qualitative and quantitative agronomic traits of significant crops to sustain crop productivity in a rapidly changing global climate. Agronomic strategies including nutrient management, microbial inoculation, plant growth regulation, and innovative agricultural technologies play an essential role in mitigating the detrimental effects of heat stress. However, future studies are urgently needed to understand the mechanisms behind heat stress reduction through microbial treatments. All these efforts will undoubtedly help to mitigate the negative effects of heat stress and contribute to improved plant productivity and food security under current scenarios of climate change and global warming.

## Author Contributions

MA generated the idea and made the 1st draft of the manuscript. All the other authors listed have made a substantial, direct, and intellectual contribution to improve the work and approved it for publication.

## Conflict of Interest

The authors declare that the research was conducted in the absence of any commercial or financial relationships that could be construed as a potential conflict of interest.

## Publisher's Note

All claims expressed in this article are solely those of the authors and do not necessarily represent those of their affiliated organizations, or those of the publisher, the editors and the reviewers. Any product that may be evaluated in this article, or claim that may be made by its manufacturer, is not guaranteed or endorsed by the publisher.
